# Premature renal epithelial cell senescence promoted by LXN/Rps3/p53 signaling pathway activation increases calcium oxalate crystal deposition by altering macrophage polarization

**DOI:** 10.3389/fimmu.2025.1658989

**Published:** 2025-10-02

**Authors:** Maolin Chu, Suna Jiang, Jiawei Xue, Wenjing Li, Guanhua Jing, Hongying Li, Juan Zhang, Wanhai Xu

**Affiliations:** 1 Department of Urology, The Second Affiliated Hospital, Harbin Medical University, Harbin, China; 2 Department of Rheumatology, The First Affiliated Hospital, Harbin Medical University, Harbin, China; 3 Department of Urology, Henan Provincial People’s Hospital (People’s Hospital of Zhengzhou University), Zhengzhou, China

**Keywords:** nephrolithiasis, cellular senescence, calcium oxalate, LXN, Rps3

## Abstract

**Introduction:**

Premature senescence of renal tubular epithelial cells (RTECs) can be caused by oxidative stress related to calcium oxalate (CaOx) kidney stones (KSs), but the role and mechanisms of cellular senescence of RTECs in the pathogenesis of kidney stones have not been fully determined. Macrophages, the most prevalent leucocyte found in nephrolithiasis, have been implicated in the pathogenesis of kidney stones.

**Methods:**

Using oxalate (Ox) induction to simulate the hyperoxaluria microenvironment in vivo, fisetin was administered to human renal proximal tubular epithelial cells (HK-2 cells). The senescence of HK-2 cells was evaluated by detecting SA-β-gal staining, expression of senescence markers p16 and p53, and levels of senescence-associated secretory phenotype (SASP) molecules. THP-1 cells were differentiated into macrophages (M0-MΦs) using PMA induction, and macrophages in different polarization states (M1-like phenotype, M2-like phenotype) were treated with the supernatant from HK-2 cell culture. siRNA gene knockdown technology was applied to evaluate the activity of the LXN/Rps3/p53 pathway during oxalate-induced senescence in HK-2 cells. A rat model of calcium oxalate crystal-induced kidney injury was established, and the rats were divided into following groups: PBS, oxalate, oxalate + fisetin, oxalate + transfection with LXN-knockdown adeno-associated virus (AAV-shLXN), and oxalate + fisetin + AAV-shLXN, Histological assessment was performed using HE staining and Von Kossa staining of kidney tissues. The expression levels of LXN, Rps3, p53, iNOS, and CD163 in renal tissues were evaluated by immunohistochemical staining.

**Results:**

The onset of RTEC senescence was increased after treatment with oxalate, and the increase in RTEC senescence was reduced by fisetin treatment. Interestingly, the changes in proinflammatory M1-like phenotype polarization induced by culture medium from HK-2 cells treated with Ox+/-fisetin were consistent with the proportion of senescent HK-2 cells cultured. Furthermore, reducing cellular LXN/Rps3/p53 signaling significantly decreased SASP factors in the culture medium and simultaneously abolished M1-like phenotype macrophage polarization. More importantly, silencing renal LXN reduced RTEC senescence and M1-like phenotype macrophage polarization and consequently decreased intrarenal CaOx crystal deposition in a rat kidney stone model.

**Discussion:**

Our results demonstrate that kidney macrophage phenotype changes are related, at least in part, to RTEC senescence, and a strategy to modulate the cellular senescence of RTECs is promising as a new target for immunotherapy to treat nephrolithiasis and other age-related diseases.

## 1 Introduction

Nephrolithiasis is one of the most common urologic ailments and poses a significant health care burden (1). Owing to its high incidence and recurrence rate (1) as well as its unclear pathogenesis, investigations of the intrinsic mechanisms and curative therapies are urgently needed. Most kidney stones are composed primarily of calcium oxalate (CaOx), and patients with kidney stones frequently suffer from hyperoxaluria (2). Numerous studies have indicated that damage to renal tubular epithelial cells (RTECs) induced by hyperoxaluria or calcium oxalate is a major factor in the formation of kidney stones (3). Hyperoxaluria and oxalate evoke oxidative stress in renal tubular cells through increased production of reactive oxygen species (ROS), which activate inflammation (3). Proinflammatory macrophages surrounding Randall’s plaques, which are recognized as the origin of calcium oxalate stone formation, have been documented in kidney tissue (4, 5). Several *in vitro (*6–8) and *in vivo (*9) studies have indicated that CaOx crystal deposition and subsequent elimination can be altered by infiltrating macrophages, which are functionally classified into two types: proinflammatory M1 and anti-inflammatory M2 macrophages. M2 macrophages can directly suppress CaOx crystal deposition by phagocytizing crystals (10, 11), whereas M1 macrophages may promote CaOx crystal deposition by altering inflammation-related oxidative stress (7, 12).

Senescence is defined as an irreversible state of cell cycle arrest that is resistant to growth-promoting stimuli (13). However, senescent cells can remain metabolically active and accumulate over time (14). Senescent cells acquire a senescence-associated secretory phenotype (SASP) (15), which converts them into proinflammatory cells that actively produce and secrete proinflammatory cytokines (16). Stress-induced premature senescence (SIPS), one of two main forms of cellular senescence, refers to the premature aging of a cell by chronic exposure to stressors (17). The major recognized inducer of SIPS is oncogenic activation under oxidative stress (18). Therefore, we hypothesized that in CaOx nephrolithiasis, oxalate and CaOx crystals induce oxidative stress and then activate a senescence gene, leading to SIPS in renal tubular cells and inflammatory SASP cytokine release (19) as well as subsequent recruitment of proinflammatory macrophages and ultimately CaOx lithogenesis. To our knowledge, no study has investigated in depth the mechanism by which cellular senescence contributes to the development of kidney stones.

Latexin (LXN), an endogenous inhibitor of metallocarboxypeptidases (20), has emerged as a multifaceted regulator in cellular processes, including inflammation (21), differentiation (22), and cancer (23). Recent studies highlight its involvement in cellular senescence in Alzheimer’s models (24), cardiovascular system (25), and cancer context (26, 27). However, whether LXN could reduce kidney stone formation through its anti-cellular senescence effects, as well as the underlying mechanisms, remains unreported in the context of nephrolithiasis. Latexin (LXN) not only inhibits metallocarboxypeptidases but also exhibits carboxypeptidase-independent roles in ribosome biogenesis (21)and stress signaling (28). It localizes in cytoplasm and nucleus, and was reported to regulate stability of ribosomal protein subunit 3 (Rps3) via direct binding (21). Moreover, Rps3 could cooperate with p53 (29), and further modulate activity of p53, which is a central hub activated by ribosomal stress to induce cell cycle arrest, senescence, or apoptosis (29). Therefore, the LXN–Rps3–p53 axis integrates ribosomal function with stress responses, and has a great potential for offering novel targets for cancer and aging interventions.

In this study, fisetin, a classic senolytic agent (30), was employed in the experiments for evaluating the impact of anti-senescence effects on aberrant macrophage polarization and subsequent stone formation. Furthermore, we screened the key gene LXN, which plays a vital role in triggering RTEC SIPS induced by oxalate. In addition, we studied the secondary events induced by senescent RTECs and the recruitment and polarization of macrophages, which play important roles in CaOx lithogenesis. More importantly, we used *in vitro* assays as well as an *in vivo* rat model to demonstrate that targeting the LXN gene in renal tubular cells could inhibit subsequent M1 macrophage polarization to eliminate CaOx crystal deposition.

## 2 Materials and methods

### 2.1 Cell culture and treatment

The HK-2 cell line (ShangHai FuHeng BioLogy, FH0228) was maintained in Dulbecco’s modified Eagle’s medium (HyClone Laboratories) supplemented with 10% FBS. HK-2 cells were treated with oxalate (Rhawn, China) or oxalate + fisetin (Aladdin, China) for 48 h. The culture medium of the oxalate (0.5 mM) or oxalate (0.5 mM)+ fisetin (10 μM) groups was collected (CM-2D) and replaced with fresh medium without oxalate or fisetin, and then HK-2 cells were further cultured for another 48 h; the culture medium (CM-4D) was collected for subsequent experiments in which macrophage-like M0 cells (M0-MΦs) were cultured. The THP-1 cells (iCell-h213) were cultured in 6-well plates with RPMI-1640 media supplemented with 10% FBS and 0.05 mM β-mercaptoethanol. The THP-1 cells were differentiated into M0-MΦs by treatment with 100 ng/ml PMA (Sigma) for 3 d (31). Adherent cells were further incubated with fresh medium containing LPS (100 ng/ml, Sigma) (32) and IFN-γ (50 ng/ml, Peprotech, Rocky Hill, USA) (33) for 24 h to stimulate M1 macrophage (M1-MΦ) polarization or with IL-4 (20 ng/ml, Peprotech, Rocky Hill, USA) (34) and IL-13 (20 ng/ml, Peprotech, Rocky Hill, USA) (35) for 24 h to stimulate M2 macrophage (M2-MΦ) polarization. Simultaneously, the aforementioned CM-4D from HK-2 cell culture was added. Macrophage polarization was assessed via RT–qPCR and western blot analyses. CM-4D and CM-2D were frozen at -20 °C until analysis using commercial enzyme-linked immunosorbent assay (ELISA) kits (Elabscience Biotechnology Co., Ltd., Wuhan, China) for IL-6, IL-1β, matrix metalloproteinase-3 (MMP-3) and MMP-13.

To knock down LXN or Rps3 expression, HK-2 cells were seeded overnight and incubated until they reached 60–80% confluence. HK-2 cells were transfected with LXN or Rps3 siRNA (Sigma–Aldrich) using Lipofectamine 3000 (Invitrogen, USA). The relative sequences are shown in **Supplementary Table 1**. During 48 h of transfection, HK-2 cells were simultaneously incubated with oxalate and then replaced with fresh culture medium for an additional 2 d of incubation; the obtained supernatant (CM-4D) was added to M0-MΦs to perform polarization experiments. For the experimental group receiving combined treatment, HK-2 cells were transfected with LXN knockdown vectors and concurrently incubated with oxalate and fisetin for 48 hours. Cellular senescence was evaluated using classic molecular markers and cell cycle regulators, including p16 and p53 (36). p16 is a critical inhibitor of CDK4/6 kinase activity that induces hypophosphorylation and sustained activation of the retinoblastoma (Rb) (37), which ultimately leads to cell cycle arrest and prevention of cell division (38). p53 is a master regulator orchestrating DNA damage responses and senescence induction (39, 40). In the group receiving combined LXN knockdown and fisetin treatment, senescence markers in HK-2 cells were assessed through SA-β-gal staining and p16 protein expression analysis.

### 2.2 SA-β-gal analysis

HK-2 cells were fixed in 4% paraformaldehyde for 10 min and then stained with a Senescence β-Galactosidase Staining Kit (Abbkine, Wuhan, China). The images were observed using microscopy (Leica DMi8).

### 2.3 Proliferation assay

HK-2 cells were seeded into 96-well plates at 5 × 10^3^ cells per well. After 48 h of incubation, HK-2 cell proliferation was detected with a cell counting kit-8 (CCK8, Multi Sciences Ltd., Hangzhou, China). The OD450 absorbance was measured to evaluate cell activity.

### 2.4 Cell biochemistry assay

The levels of superoxide dismutase (SOD), total antioxidant capacity (T-AOC), glutathione (GSH) and lactate dehydrogenase (LDH) were measured using commercial assay kits from Nanjing Jiancheng Bioengineering Institute of China (SOD, A001-3-2; T-AOC, A015-2–1; GSH, A006-2-1; and LDH, A020-2-2) according to the manufacturer’s instructions.

### 2.5 Immunocytofluorescence staining

Cells were incubated with 0.5% Triton X-100 (MERCK) for 20 min and washed with PBS. Nonspecific binding was blocked by incubation with normal horse serum (Gibco) at 37 °C for 30 min. The cells were then incubated with anti-p16 antibody (Abcam, ab108349) or anti-Rps3 antibody (Abcam, ab128995) at 4 °C overnight, followed by incubation with an Alexa Fluor 488-conjugated goat anti-rabbit IgG secondary antibody (AmyJet Scientific Inc., Wuhan, China) in the dark at 37 °C for 1 h. Coverslips with stained cells were further stained with DAPI (Abcam). Positive cells were visualized and imaged using a fluorescence microscope (Leica DMi8).

### 2.6 Quantitative real-time PCR and western blotting

Cellular RNA extraction, reverse transcription, and RT–qPCR were performed as described previously (41). The transcript levels were normalized to those of GAPDH. Gene expression was calculated using the 2-ΔΔCt method. The specific primers used are shown in **Supplementary Table 1**.

Protein preparation and western blot analysis were performed as described previously (42, 43). The primary antibodies used were as follows: anti-VEGF rabbit antibody (Abcam, ab52917), anti-MMP13 rabbit antibody (Abbkine, Wuhan, China, ABP51805), anti-IL-1β rabbit antibody (Abcam, ab254360), anti-MCP1 rabbit antibody (Abcam, ab214819), anti-LXN rabbit antibody (Abcam, ab154744), anti-Rps3 rabbit antibody (Abcam, ab128995), anti-p16 rabbit antibody (Abcam, ab108349), anti-p53 rabbit antibody (Abcam, ab32389), anti-TNF-α rabbit antibody (Abbkine, Wuhan, China, ABM0127), anti-iNOS rabbit antibody (Abcam, ab283655), anti-Arg-1 rabbit antibody (Abcam, ab96183) and anti-CD163 rabbit antibody (Abcam, ab182422). β-actin or GAPDH was used for normalization.

### 2.7 Animal studies

All experimental procedures were performed following the rules of the National Institutes of Health Guide for the Care and Use of Laboratory Animals, and approval was obtained from the Ethics Committee on the Use of Live Animals of Harbin Medical University. Male Sprague–Dawley rats (180–220 g, 6–8 weeks old) were randomly divided into six groups: the control group, oxalate group, oxalate+fisetin group, oxalate+AAV-Null group and oxalate+AAV-shLXN group,oxalate + fisetin + AAV-shLXN group. Normal control rats were given an equal volume of PBS. The rats received intraperitoneal injection of 60 mg/kg glyoxylic acid (Sigma–Aldrich) five times a week for four weeks (oxalate group) (44) to establish the kidney stone rat model. Fisetin, a classic senolytic, was intragastrically administered at 50 mg/kg to kidney stone model rats five days a week for four weeks (45)from the first day of glyoxylate injection. To knockdown LXN expression *in vivo*, rats were transduced with an AAV serotype 9 vector encoding a green fluorescent protein reporter together with either short hairpin RNAs (shRNAs) targeting LXN in the kidney (AAV-shLXN) or an empty vector (AAV-null) (Shanghai GeneChem Co., Ltd. Shanghai, China). The rats were injected with either AAV-shLXN or AAV-Null via the tail vein, followed by an additional glyoxylate injection (oxalate+AAV-shLXN group). The rats treated with glyoxylate and AAV-Null vector injection (oxalate + AAV-Null group) were used as the virus transfection control group. All animal kidney tissues were obtained on Day 30 after the first injection of glyoxylate.

### 2.8 Histology and immunohistochemical staining

Rat kidneys were embedded in paraffin, and the samples were sectioned. Hematoxylin and eosin (HE) staining, Von Kossa staining and immunohistochemical analysis were performed as described previously (46–49).Immunostaining was performed with specific antibodies against the target proteins LXN (Bioss, bs-1971R), Rps3 (Absin, abs116130), p53 (Bioss, bs-2090R), p16 (Bioss, bs-0740R), iNOS (Bioss, bs-0162R) and CD163 (Bioss, bs-2527R). Images were captured using a microscope (Leica DMi8). Kidney injury scores were evaluated using a scale ranging from 0 to 4 as described before (50). Briefly, 0 indicating normal; 1 representing less than 25%; 2 indicating 25-50%; 3 representing 50-75%; and 4 indicating greater than or equal to 75%. Quantitative evaluation of Von Kossa staining and immunohistochemical analysis were performed using Image J software as described previously (51).

### 2.9 Statistical analysis

Statistical analysis was performed using IBM SPSS 27.0. Data were presented as the mean ± Standard Deviation (SD). Two-tailed Student’s t-tests and one way ANOVA followed by Tukey’s test. were used for normally distributed data when appropriate. A non-parametric test was used for non-normally distributed data. *P* < 0.05 was considered statistically significant.

## 3 Results

### 3.1 Fisetin alleviated oxalate-induced injury to RTECs via its anti-senescence effect

**Figure 1:**
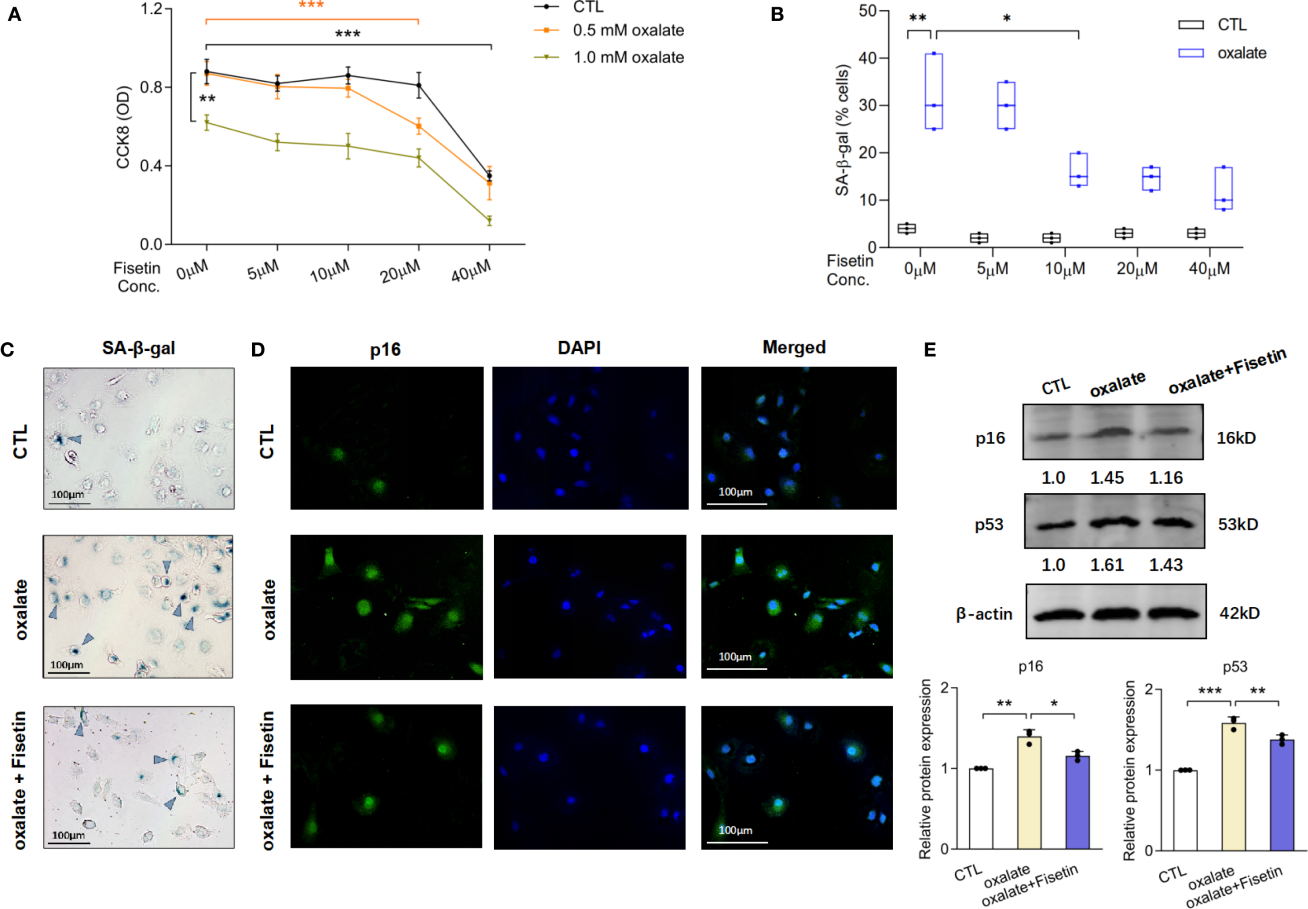
Fisetin mitigated oxalate-induced injury of HK-2 cells via its anti-senescence and anti-oxidative stress effect. **(A)** HK-2 cells were incubated under different concentrations of fisetin and oxalate for 48h. CCK-8 assay was used to detect cell viability. 0.5 mM oxalate and concentrations of fisetin (5 and 10 μM) have no significant effect on cell viability of HK-2 cells. **(B)** SA-β-gal staining in HK-2 cells treated with the combination of 0.5 mM oxalate and different concentrations of fisetin for 48h were performed. Frequency of SA-β-gal (+) senescent cells increased significantly after oxalate incubation, and furthermore, 10 μM fisetin could significantly decrease the proportion of SA-β-gal (+) cells induced by oxalate compared with dose of 5 μM fisetin. And **(C)** typical pictures of SA-β-gal staining of HK-2 cells under incubation of oxalate with/without 10 μM fisetin were shown. **(D)** Immunofluorescence detection demonstrated that the changing trends of p16 staining intensity were in accordance with the alteration of SA-β-gal (+) senescent cells’ frequency. **(E)** Western blot analysis showed that relative p16 and p53 expression in HK-2 were significantly increased by 0.5 mM oxalate, and this elevation could be partially reversed by 10 μM fisetin. Based on three independent experiments. Data are presented as means ± SD. *P < 0.05, **P < 0.01, ***P < 0.001; CTL, control group.

Fisetin, a classic senolytic agent, was employed in this study to treat cells for evaluating the impact of anti- senescence effect on aberrant macrophage polarization and subsequent stone formation. To determine the effects of oxalate with or without fisetin on cell viability, HK-2 cells were treated with oxalate (0, 0.5 or 1.0 mM) and varying concentrations of fisetin (0, 5, 10, 20 or 40 μM) for 48 h. HK-2 cell viability was significantly lower at a dose of 1.0 mM oxalate compared with that of the vehicle control. Incubating HK-2 cells with 0.5 mM oxalate and various concentrations of fisetin (5 and 10 μM) had no significant effect on cell viability. Incubating HK-2 cells with 40 μM fisetin or 0.5 mM oxalate+ 20 μM fisetin significantly decreased cell viability compared with that of the control group (**Figure 1A**). Since substantial viability drops demonstrated the cytotoxic potential of oxalate and fisetin, the maximum non-cytotoxic doses (just before the significant viability decrease) from agent’s concentration gradient were selected for subsequent cellular senescence experiments. Substantial numbers of senescent cells were evaluated via senescence-associated β-galactosidase (SA-β-gal) activity, and the results revealed that the frequency of SA-β-gal(+) senescent cells increased significantly after 0.5 mM oxalate treatment, and that 10 μM fisetin significantly decreased the proportion of SA-β-gal (+) senescent cells (**Figure 1B**). Typical images are shown in **Figure 1C**. Therefore, we treated HK-2 cells with 0.5 mM oxalate for 48 h to establish a model of RTEC injury and 10 μM fisetin was administered simultaneously to evaluate the effect of anti-senescence in the subsequent experiments. In addition to SA-β-gal activity detection, the expression of typical markers of cellular senescence (e.g. p16 and p53) were also determined via immunofluorescence staining and western blotting. Immunofluorescence revealed that p16 staining was more intense in HK-2 cells incubated with oxalate than in control HK-2 cells, and 10 μM fisetin inhibited p16 staining intensity under oxalate induction (**Figure 1D**). Western blot analysis revealed that oxalate significantly increased the levels of p16 and p53, and fisetin reversed this effect of oxalate on p16 and p53 expression (**Figure 1E**).

**Figure 2:**
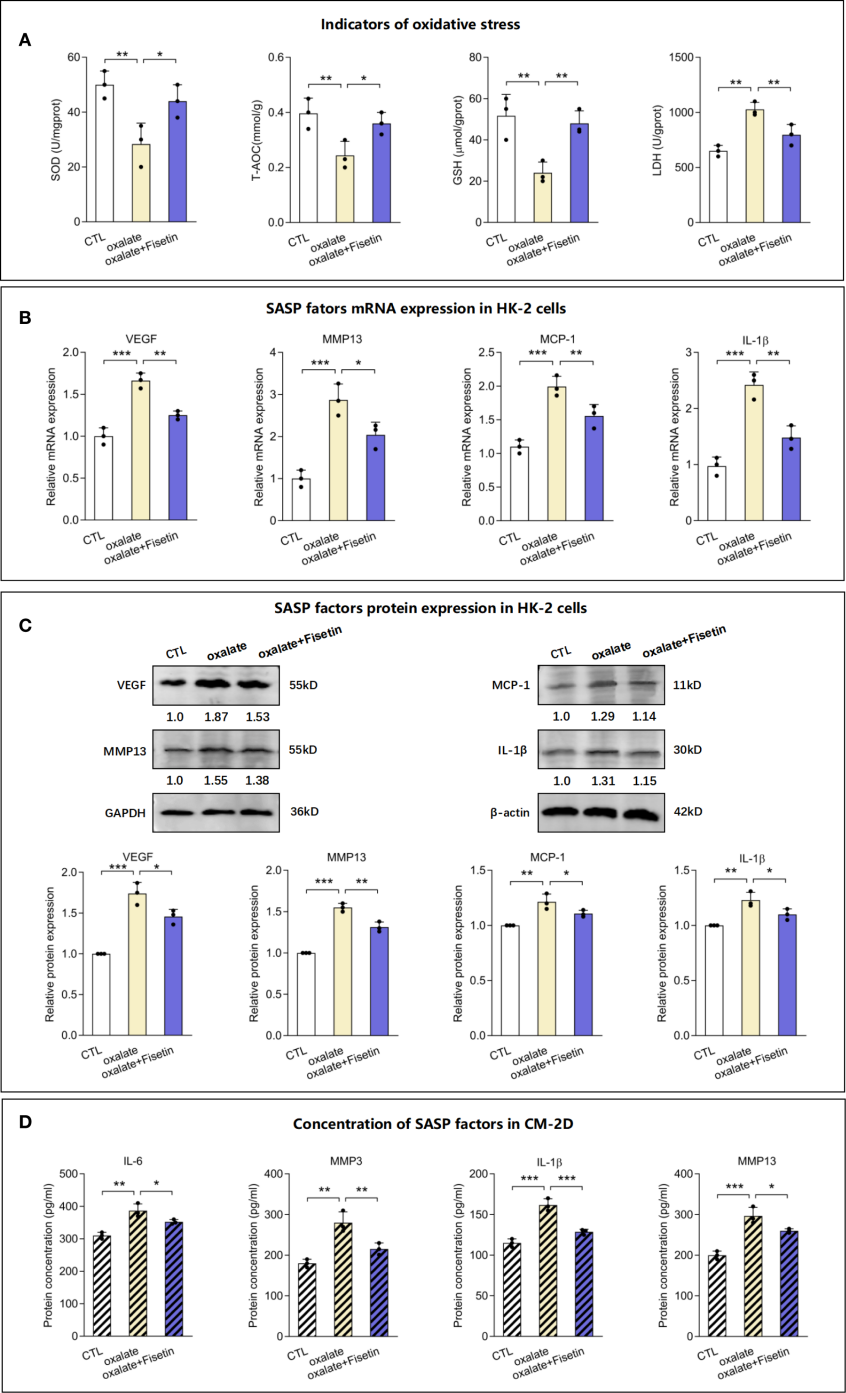
Levels of oxidative stress indicators and SASP factors in senescent HK-2 cells. HK-2 cells were treated with 10 μM fisetin in the presence of 0.5 mM oxalate for 48h and then the following indicators were determined. **(A)** Status of oxidative and anti-oxidative indicators in HK-2 cells, including SOD content, T-AOC content, GSH content and LDH content, were measured. The mRNA **(B)** and protein **(C)** levels of SASP factors, VEGF, MMP13, MCP-1 and IL-1β, in HK-2 cells were determined using RT-qPCR or western blot respectively. **(D)** Concentration of SASP factors, IL-1β, MMP13, IL-6 and MMP3, in culture medium of 2 days (CM-2D) were detected by Elisa analysis. Based on three independent experiments. Data are presented as means ± SD. *P < 0.05, **P < 0.01, ***P < 0.001; CTL, control group. CM-2D, culture medium from the 2 Day’s culture.

Superoxide dismutase (SOD), an antioxidant enzyme, catalyzes the decomposition of superoxide radicals into oxygen and hydrogen peroxide, thereby reducing oxidative stress damage. Total antioxidant capacity (T-AOC) can be used to evaluate the overall antioxidant level of all antioxidant substances (including enzymatic and non-enzymatic antioxidants) in samples such as serum. Glutathione (GSH) is a critical non-enzymatic antioxidant within cells that directly scavenges free radicals and participates in maintaining redox homeostasis. Lactate dehydrogenase (LDH) is a key enzyme in glycolysis that catalyzes the interconversion between pyruvate and lactate. It is commonly used as a biomarker for cellular damage or death, as it is released into the bloodstream upon rupture of the cell membrane. Subsequently, the levels of the aforementioned oxidative and antioxidative indicators, including SOD, T-AOC, GSH and LDH, were measured to evaluate injury to RTECs. The levels of SOD, T-AOC and GSH were significantly lower in the oxalate group than in the control group, whereas the LDH level was higher. Compared with those of the oxalate group, the oxalate + fisetin group presented elevated SOD, T-AOC and GSH levels and reduced LDH levels (**Figure 2A**). Next, we used a model of oxalate-induced senescent HK-2 cells as described above to determine whether HK-2 cell senescence paralleled the increased release of proinflammatory factors of the senescence-associated secretory phenotype (SASP). We collected mRNA and protein from control cells and senescent HK-2 cells treated with oxalate with or without fisetin in 2-d cultures. The mRNA and protein expression of the proinflammatory SASP-associated factors VEGF, MMP13, MCP-1 and IL-1β were upregulated by oxalate, and this upregulation was partially reversed by fisetin (**Figures 2B, C**). The changes in the concentrations of the SASP-associated factors IL-1β, MMP13, IL-6 and MMP3 in the culture medium from 2-day-old cultures (CM-2D) were similar to the MMP13 and IL-1β expression in control cells (**Figure 2D**). On the third day, the supernatants of HK-2 cells induced with oxalate with or without fisetin were replaced with fresh culture medium, and 2 days later, the culture medium (CM-4D) was obtained for subsequent experiments on macrophage polarization to exclude the effects of oxalate or fisetin on the biological behavior of macrophages. IL-1β, MMP13, IL-6 and MMP3 levels of CM-4D from HK-2 cell cultures after oxalate induction were significantly upregulated, and this upregulation was also significantly decreased in the CM-4D from oxalate+fisetin-treated HK-2 cell cultures (**Supplementary Figure 1**).

### 3.2 Senescent HK-2 cells played a significant role in macrophage polarization

**Figure 3:**
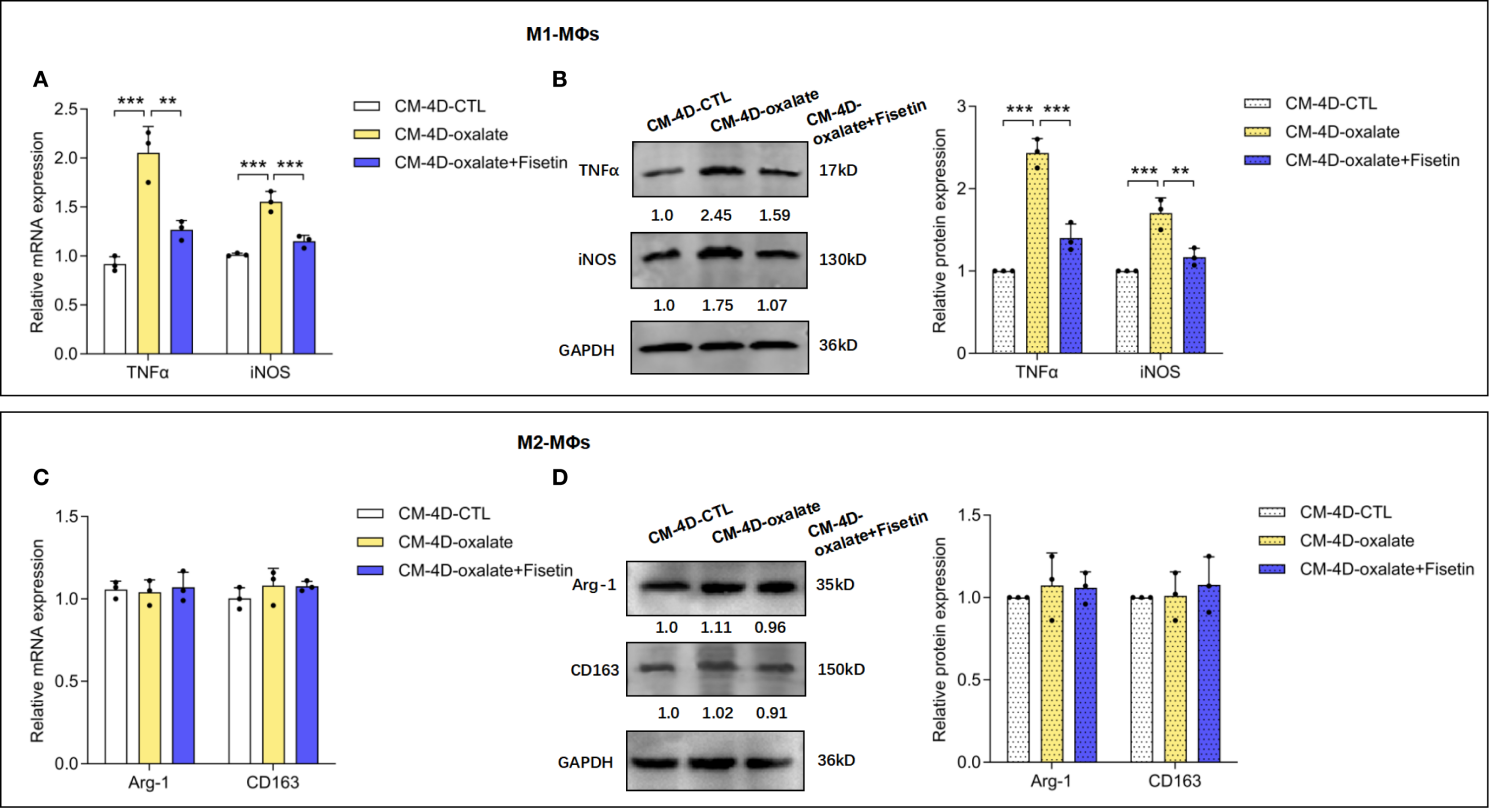
Senescence of HK-2 cells shifted macrophage polarization. HK-2 cells were treated with oxalate or oxalate + fisetin for 48 h. The culture medium were removed with fresh medium, and then HK-2 cells were further cultured for another 48h thus the culture medium (CM-4D) were collected. THP-1 cells were induced towards macrophage differentiation by PMA (100 ng/ml), and macrophages (MΦs) were further incubated with CM-4D from HK-2 cells after treatment of oxalate or oxalate+fisetin under LPS (100 ng/ml) & IFN-γ (50 ng/ml) or IL-4 (20 ng/ml) & IL-13 (20 ng/ml) stimulatory conditions for 24 h. MΦs differentiated either to M1 **(A, B)** or M2 **(C, D)** macrophages were further analyzed by RT-qPCR **(A, C)** or western blot **(B, D)**. **(A, B)** CM-4D from oxalate induced HK-2 cells (CM-4D-oxalate) could significantly increase TNF-α and iNOS mRNA **(A)**/protein **(B)** expression in M1-MΦs, and this up-regulation could be significantly reversed by CM-4D from oxalate+fisetin treated HK-2 cells (CM-4D-oxalate+fisetin). **(C, D)** There was no significant change of Arg-1 and CD63 mRNA **(C)**/protein **(D)** expression in M2-MΦs under management of CM-4D from oxalate +/- fisetin treated HK-2 cells. Based on three independent experiments. Data are presented as means ± SD. **P < 0.01, ***P < 0.001; CTL, control group.

To study whether senescence of RTECs can influence the polarization of M0-MΦs into either inflammatory macrophages (M1-MΦs) or anti-inflammatory macrophages (M2-MΦs), THP-1 cells were induced to differentiate into macrophages via PMA treatment. Based on IFNγ and LPS induction toward M1-MΦs or IL-4 and IL-13 induction toward M2-MΦs for another 24 h, we assayed macrophage polarization in the culture medium (CM-4D) from HK-2 cells treated with oxalate or oxalate+fisetin. In current macrophage research, TNF-α (52) and iNOS (53) are well-established biomarkers for identifying M1 macrophages, whereas Arg-1 (54) and CD163 (55) are widely recognized as markers of M2 macrophages (56). Therefore, in accordance with conventional macrophage classification schemes (57, 58), we employed TNF-α and iNOS for the identification of M1 macrophages and used Arg-1 and CD163 to characterize M2 macrophages in this study. As shown in **Supplementary Figure 2**, compared with those in native macrophages, the mRNA expression of TNF-α and iNOS was increased in M1 macrophages, and the expression of Arg-1 and CD163 mRNA was increased in M2 macrophages, confirming the success of macrophage induction. As shown in **Figures 3A, B**, CM-4D from oxalate-induced HK-2 cells (CM-4D-oxalate) significantly increased TNF-α and iNOS mRNA/protein expression in M1-MΦs, and this upregulation was significantly reversed by CM-4D from oxalate+fisetin-treated HK-2 cells (CM-4D-oxalate+fisetin). However, there was no significant change in Arg-1 or CD163 mRNA/protein expression in M2-MΦs in response to CM-4D from oxalate +/- fisetin-treated HK-2 cells (**Figures 3C, D**). These results indicated that CM-4D oxalate induced the polarization of M0-MΦs toward M1-MΦs but not toward M2-MΦs.

### 3.3 LXN played an important role in oxalate-mediated promotion of HK-2 cell senescence and subsequent macrophage polarization

**Figure 4:**
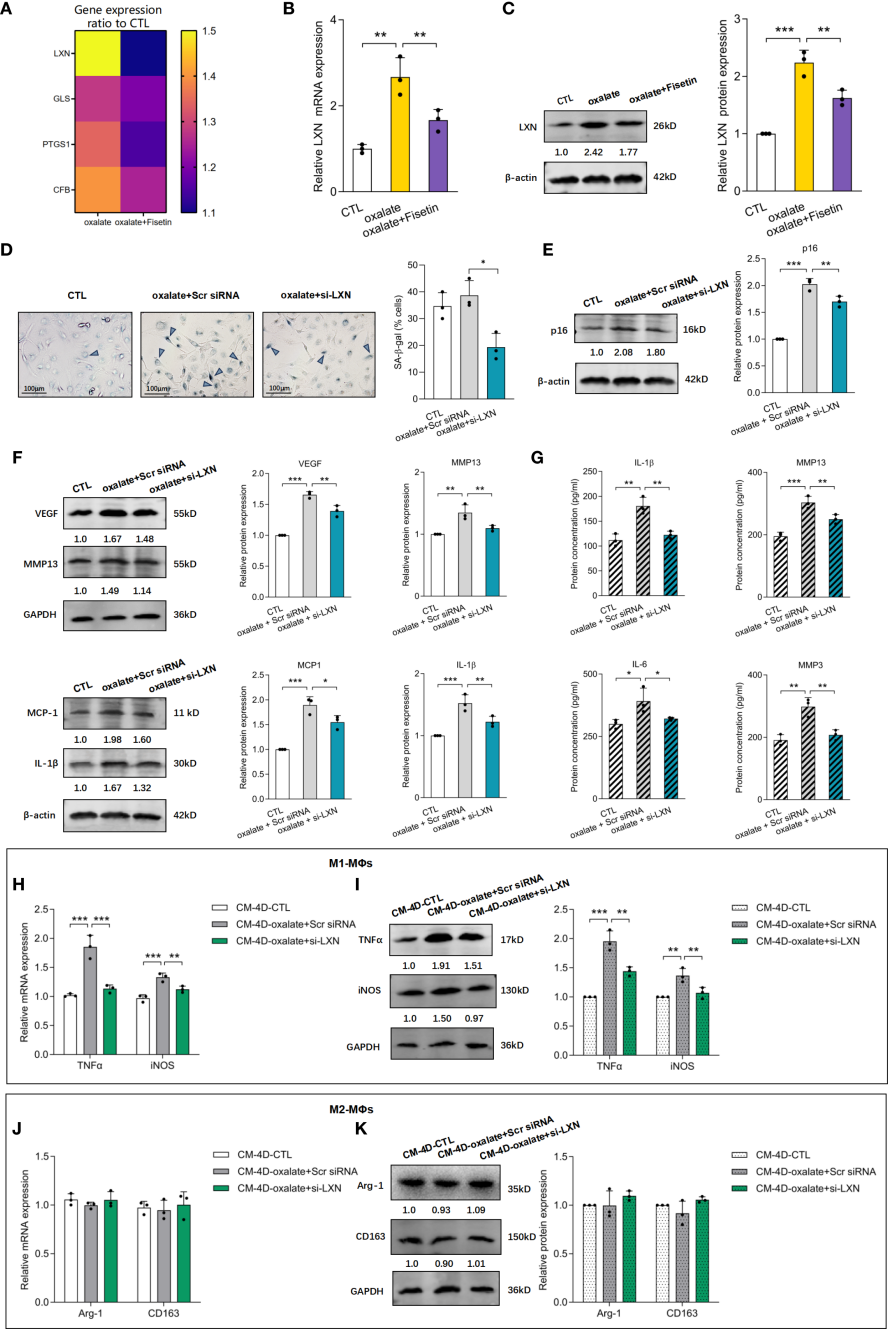
LXN knockdown inhibited oxalate induced senescence of HK-2 cells as well as subsequent macrophage polarization. HK-2 cells were treated with 0.5 mM oxalate with/without 10 μM fisetin. **(A)** LXN, GLS, PTGS1 and CFB mRNA expression were determined via RT-qPCR. The ratio of relative expression of LXN, GLS, PTGS1 and CFB genes induced by oxalate or oxalate+fisetin compared to control group were shown. Relative LXN mRNA levels detected via RT-qPCR **(B)** and LXN protein expression determined via western blot **(C)** were significantly increased in oxalate induced HK-2 cells compared to control cells, and further significantly down-regulated by fisetin. LXN knockdown was performed via transfecting specific LXN small interfering RNA (siRNA) into HK-2 cells, and these HK-2 cells were further incubated with/without oxalate for 48h. Analysis of senescence in HK-2 cells were performed via SA-β-gal activity **(D)** and relative p16 protein expression **(E)**. Results showed that SA-β-gal activity and p16 protein level were significantly increased by oxalate, and further significantly decreased under LXN knockdown intervention. SASP-associated factors **(F)** (VEGF, MMP13, MCP-1 and IL-1β) in HK-2 cells and **(G)** (IL-1β, MMP13, IL-6 and MMP3) in HK-2 cells culture medium demonstrated similar changing trends with p16 protein levels. THP-1 cells were stimulated with PMA for differentiation into macrophages (MΦs). Polarization was performed with LPS & IFN-γ (M1) or IL-4 & IL-13 (M2) for 24 h, and simultaneously, culture medium from fresh medium incubated HK-2 cells for another 48h which cells have been already knocked down by si-LXN with/without oxalate intervention for 48h. THP-1 cells differentiated either to M1 **(H, I)** or M2 **(J, K)** macrophages were analyzed by RT-qPCR **(H, J)** or western blot **(I, K)**. Based on three independent experiments. Data are presented as means ± SD. *P < 0.05, **P < 0.01, ***P < 0.001; CTL, control group. Scr siRNA, Scrambled siRNA control.

Compared with those in healthy controls, the expression of four senescence-associated genes, including LXN, GLS, PTGS1 and CFB, in nephrolithiasis patients (59) was found to be significantly upregulated in HK-2 cells treated with oxalate with or without fisetin (10 μM). RT–qPCR revealed that the LXN, GLS, PTGS1 and CFB genes were expressed at distinctly higher levels in oxalate-induced HK-2 cells than in control cells and were further significantly downregulated by fisetin. The ratios of the relative expression of the above genes compared with those of the control group are shown in **Figure 4A**, and the relative LXN mRNA levels are shown in **Figure 4B**. LXN, the gene expression of which varied most significantly after oxalate + fisetin intervention, was selected for further study. The protein expression of LXN after oxalate treatment with or without fisetin was confirmed to have a similar effect on gene expression (**Figure 4C**). To further evaluate the regulatory effect of LXN on cellular senescence, experiments with LXN knockdown in HK-2 cells were carefully performed, and the alterations in biological behaviors related to cellular senescence were evaluated. The knockdown efficiency of LXN was determined by RT–qPCR and western blot analyses. At 48 h after transfection, 75% knockdown efficiency was achieved, as shown by both gene and protein expression (**Supplementary Figures 3A, B**). The results of senescence-associated β-galactosidase (SA-β-gal) activity experiments showed that the increased proportion of SA-β-gal (+) cells induced by oxalate was significantly decreased by LXN knockdown (**Figure 4D**). In addition, LXN knockdown significantly decreased the protein expression of p16 and SASP-associated factors (VEGF, MMP13, MCP-1 and IL-1β), which were dramatically increased in oxalate-induced HK-2 cells (**Figures 4E, F**). Consistent with changes in the protein expression of SASP-associated factors in HK-2 cells, the concentrations of SASP factors (i.e., IL-1β, MMP13, IL-6 and MMP3) in the supernatant of HK-2 cell cultures demonstrated similar trends after oxalate treatment with or without LXN knockdown intervention (**Figure 4G**). Interestingly, the group treated with LXN knockdown combined with fisetin showed a significant reduction in both the proportion of SA-β-gal (+) cells and the protein expression of p16 compared to the groups treated with LXN knockdown or fisetin alone (**Supplementary Figure 4**). The combined treatment of fisetin and LXN knockdown reduced the levels of senescence markers to an extent that was nearly additive but not fully linear, suggesting the effect of LXN occurs through the fisetin–senescence pathway.

To observe the influence of delaying senescent HK-2 cell accumulation induced by LXN knockdown on macrophage polarization, HK-2 cells subjected to LXN knockdown were simultaneously incubated with oxalate, which was replaced with fresh culture medium for an additional 2 d, after which the supernatants were obtained (CM-4D) and added to PMA + IFNγ + LPS- or PMA + IL-4 + IL-13-stimulated THP-1 cells. As shown in **Figures 4H, I**, CM-4D from oxalate-induced scramble siRNA-transfected HK-2 cells (CM-4D-oxalate+Scr siRNA) significantly increased TNF-α and iNOS mRNA/protein expression in M1-MΦs, and this upregulation was significantly reversed by CM-4D from oxalate-treated HK-2 cells after LXN knockdown (CM-4D-oxalate+si-LXN). Furthermore, there was no significant change in Arg-1 or CD163 mRNA/protein expression in M2-MΦs treated with CM-4D from HK-2 cells exposed to oxalate+Scr siRNA or oxalate+si-LXN compared with those in the control group (**Figures 4J, K**). These results suggest that M1-MΦ polarization induced by culture medium from HK-2 cells under oxalate+LXN-knockdown (CM-4D-oxalate+si-LXN) intervention was significantly lower than that induced by CM-4D-oxalate+Scr siRNA. Overall, we concluded that LXN knockdown effectively attenuated the oxalate-induced senescent response and subsequently attenuated proinflammatory M1 macrophage polarization.

### 3.4 LXN knockdown reduced senescent cell burden by increasing nuclear translocation of Rps3 and subsequent p53 reduction

**Figure 5:**
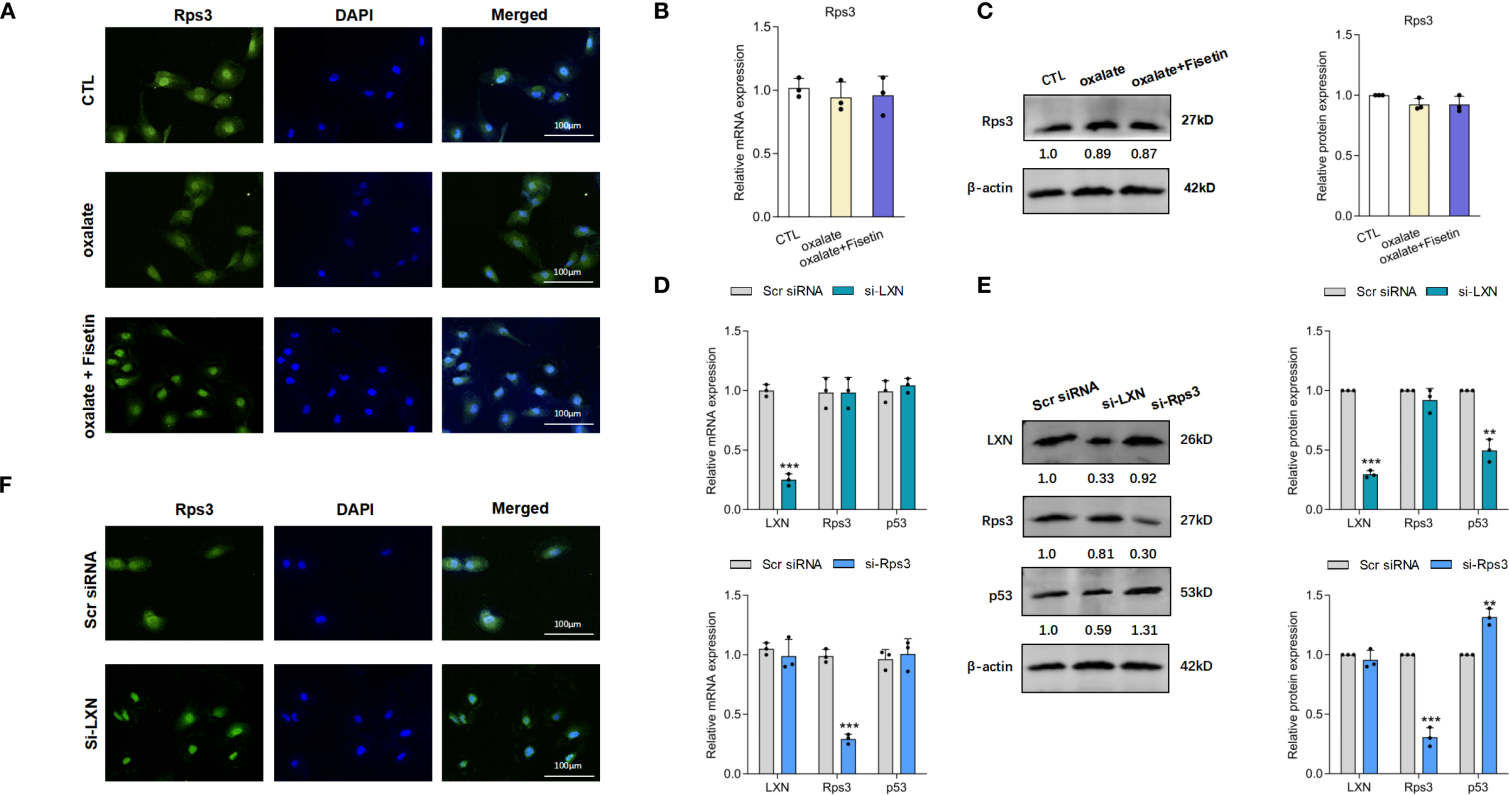
LXN/Rps3/p53 pathway was involved in the oxalate induced senescent cell burden. **(A)** Laser confocal microscopy was applied to evaluate nuclear localization of Rps3. Nuclear localization of Rps3 were significantly decreased in HK-2 cells under oxalate induction compared to control cells, and these decreased nuclear translocation of Rps3 were significantly up-regulated by fisetin treatment. mRNA **(B)** and protein **(C)** expression of Rps3 in HK-2 cells under oxalate with/without fisetin treatment appeared no significant changes compared to control group. Oxalate induction was performed on the basis of LXN knockdown. **(D)** LXN knockdown has little effect on expression levels of Rps3/p53 mRNA, and Rps3 silencing has little effect on LXN3/p53 mRNA expression. **(E)** p53 protein expression was significantly down-/up-regulated by LXN/Rps3 knockdown. **(F)** Nuclear translocation of Rps3 was obviously elevated after LXN silencing. Data are presented as means ± SD. **P < 0.01, ***P < 0.001; CTL, control group. Scr siRNA=Scrambled siRNA control.

Because LXN has been reported to perform its biological function by binding to the protein ribosomal protein 3 (Rps3) (60) and Rps3 has an important extraribosomal role in the DNA damage response and in the regulation of p53 degradation (61), we detected the expression of Rps3 and p53 in the next step to investigate the mechanism by which LXN inhibits the senescence of HK-2 cells. Interestingly, less Rps3 was localized to the nucleus in HK-2 cells under oxalate induction than in control cells, and the decreased nuclear translocation of Rps3 was significantly increased by fisetin (10 μM) treatment (**Figure 5A**). This finding was further confirmed by the lack of significant changes in the mRNA and protein expression of Rps3 in the presence of oxalate with or without fisetin treatment (**Figures 5B, C**). To evaluate the relationships among LXN, Rps3 and p53, LXN or Rps3 knockdown was performed. The knockdown efficiency of Rps3 is shown in **Supplementary Figures 3C, D**. As demonstrated in **Figures 5D, E**, knockdown of LXN had little effect on the expression levels of Rps3 mRNA, p53 mRNA and Rps3 protein, whereas p53 protein expression was significantly downregulated. When Rps3 was knocked down, the expression levels of LXN mRNA, p53 mRNA and LXN protein did not significantly change, whereas p53 protein expression was significantly upregulated, suggesting that p53 is downstream of LXN/Rps3 and that LXN exerts its effects in combination with Rps3 to affect the nuclear translocation of Rps3; these findings are further supported by the result that nuclear translocation of Rps3 was obviously elevated after LXN silencing, as shown by immunofluorescence experiments (**Figure 5F**).

### 3.5 Targeting cellular senescence could ameliorate renal impairment and crystal deposition in the rat kidney stone model

**Figure 6:**
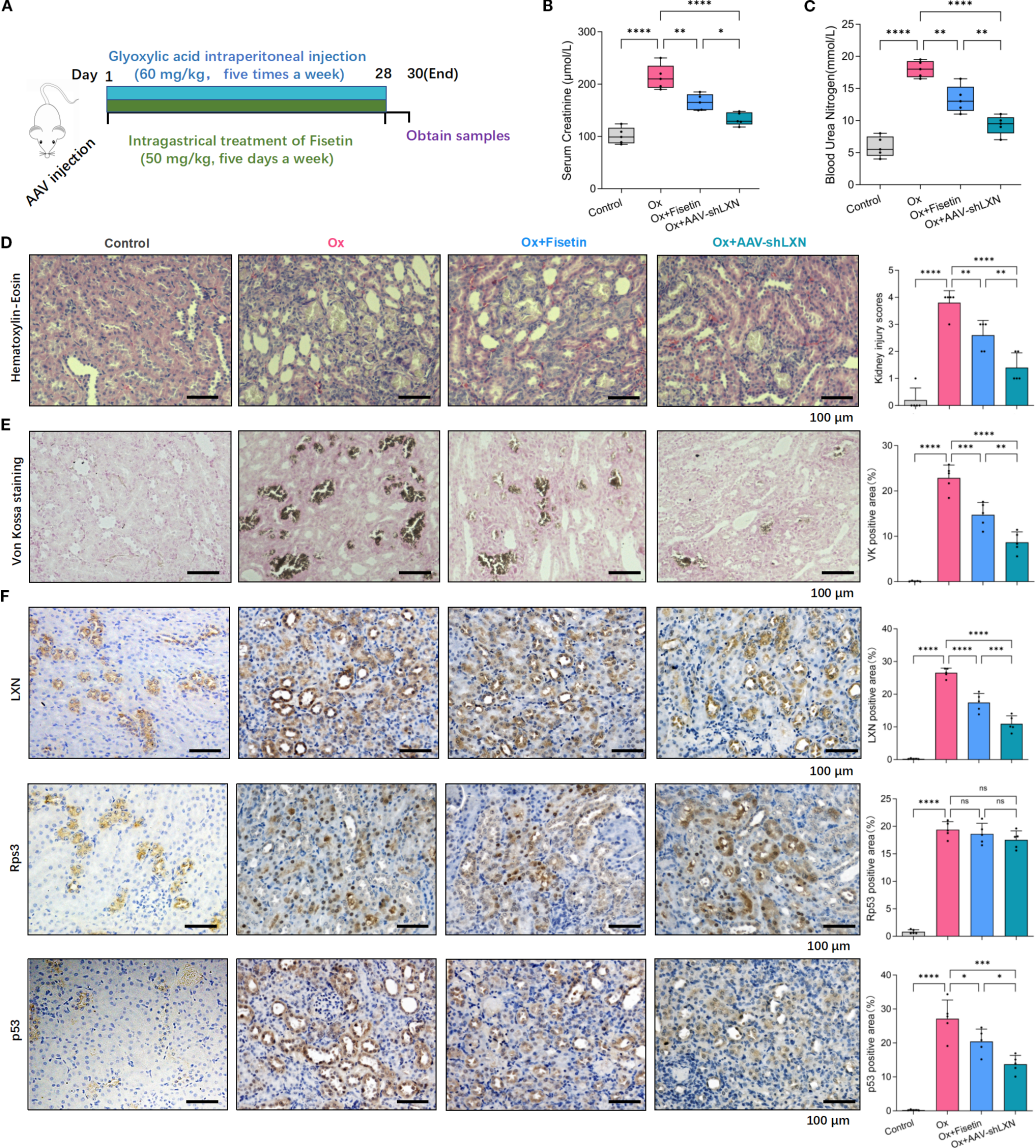
Cellular senescence-targeting therapy could ameliorate the renal impairment and crystal depositions in the animal model. **(A)** Process diagrams are demonstrated. **(B, C)** The levels of Scr **(B)** and BUN **(C)** in kidney stone model rats were measured. Compared with those in the normal group, Scr and BUN levels in the oxalate group were increased notably on Day 30, and fisetin markedly ameliorated the renal functional damage caused by oxalate crystals. The elevated levels of Scr and BUN in oxalate group could be significantly relieved by injection of AAV-shLXN. **(D)** Renal histological analyses of H&E staining showed that fisetin lowered the grade of tubules dilatation along with decreased levels of mononuclear cell infiltrates in the interstitium compared to that in oxalate group. AAV-shLXN showed a more significant inhibitory effect on tubules dilatation and mononuclear cell infiltrates compared to fisetin. **(E)** Von Kossa staining revealed similar changing trends of crystal formation in kidney tissue as histological H&E staining analysis. **(F)** Immunohistochemistry analysis demonstrated that immunostaining intensity of LXN and p53 were significantly stronger in the oxalate group compared with normal group, and the staining intensity of LXN and p53 were significantly decreased under fisetin or AAV-shLXN administration, even more significantly in AAV-shLXN group. There was no significant change of total Rps3 staining among different groups. Data are presented as means ± SD. Data are expressed as the mean ± SD. *P < 0.05, **P < 0.01, ***P < 0.001, ****P < 0.0001. “ns” indicates no significant difference.

The experimental procedures for animal studies are schematically depicted in **Figure 6A**. Blood Urea Nitrogen (BUN) is a common diagnostic readout, with higher levels indicating impaired renal function and reduced glomerular filtration rate. Serum creatinine (Scr) is also a widely used and clinically essential biomarker for evaluating renal function. Higher Scr levels typically reflect a greater degree of kidney impairment. To investigate the *in vivo* role of cellular senescence in kidney stone formation as well as renal impairment, the levels of Scr and BUN were measured. Compared with those in the normal group, the Scr and BUN levels in the oxalate group were notably increased on Day 30, and the Scr and BUN levels in the oxalate+fisetin group were markedly lower than those in the oxalate group, indicating the amelioration of renal impairment after fisetin administration (**Figures 6B, C**). Renal histological analyses showed that fisetin attenuated oxalate-induced injury to RTECs, as demonstrated by lower-grade dilatation of the tubules and decreased levels of mononuclear cell infiltrates in the interstitium of rats in the oxalate+fisetin group compared with that in the oxalate group (**Figure 6D**). Von Kossa staining of isolated kidney tissues revealed similar differences in crystal formation (**Figure 6E**), suggesting that fisetin has a protective role in ameliorating the formation of kidney stones. Furthermore, we examined the expression levels of the LXN/Rps3/p53 pathway in kidney tissue by immunohistochemistry staining. Our results showed that, compared with that under normal conditions, LXN and p53 protein expression was significantly increased in the oxalate group, whereas fisetin administration significantly decreased LXN and p53 protein expression. Compared with that in the oxalate group, the total Rps3 protein staining intensity was not significantly different, and less Rps3 protein staining was observed in the cell nuclei of the oxalate+fisetin group (**Figure 6F**). Immunohistochemical staining of the polarization biomarkers iNOS (M1-MΦs) and CD163 (M2-MΦs) was also performed to evaluate macrophage polarization, and immunohistochemical staining revealed that iNOS was significantly increased in the kidney tissue of the oxalate group. This increase in iNOS staining was significantly decreased in the oxalate+fisetin-treated group. However, there was no significant difference in CD163 staining among the control, oxalate and oxalate+fisetin groups (**Supplementary Figure 5**).

To study the effect of the LXN gene on cellular senescence in nephrolithiasis, AAV-shLXN or AAV-null vectors (as controls) were injected via the tail vein into stone model rats on the first day. Scr/BUN determinations, H&E analysis and Von Kossa staining showed that, compared with that of the stone model oxalate group, the oxalate+AAV-Null group had a similar effect on crystal deposition (data not shown). Interestingly, H&E, Von Kossa staining and immunohistochemical analysis revealed that, compared with those of the fisetin group, AAV-shLXN had a similar effect on crystal deposition, LXN/Rps3/p53 and iNOS/CD163 protein staining; more importantly, the protective effect of AAV-shLXN was even greater than that of fisetin (**Figures 6D–F**, **Supplementary Figure 5**). More importantly, the combined treatment with fisetin and LXN knockdown led to a more pronounced reduction in Scr and BUN levels, tubular dilation, mononuclear cell infiltration, crystal deposition, and expression levels of LXN, p53, and iNOS in renal tissue—as assessed by immunohistochemical staining—compared to each treatment alone (**Supplementary Figures 5**, **6**). Taken together, our *in vivo* experimental results suggest that senescence-targeting therapy effectively inhibits cellular senescence through the LXN/Rps3/p53 pathway, thus reducing macrophage polarization toward the M1 phenotype and ultimately exerting a protective effect against kidney stone formation. LXN gene silencing might be a potential therapeutic method for treating nephrolithiasis.

## 4 Discussion

Although the incidence of nephrolithiasis increases with increasing age (62, 63), and the abundance of senescent cells in various tissues increases with age (64, 65), few studies have investigated the exact relationship, particularly the mechanism involved in cellular senescence-related nephrolithiasis. First, this study reports that RTEC senescence, in addition to oxidative stress, is triggered by oxalate induction, producing a bioactive secretome and SASP factors that mediate recruitment and M1 macrophage polarization, ultimately leading to CaOx lithogenesis. Therefore, senescence could constitute a newly described mechanistic link between oxidative stress and inflammation-triggered recruitment and polarization of macrophages in CaOx lithogenesis. This result was consistent with the phenomenon of premature RTEC senescence induced by oxidative stress in the urine of patients with calcium oxalate nephropathy, which was reported by Kamonchanok Chuenwisad et al. (66). More importantly, the LXN gene was confirmed to play a vital role in triggering premature senescence in RTECs under oxalate stress by reducing the nuclear translocation of Rps3, which decreases p53 expression. Therefore, LXN could be a novel molecular target to improve the prevention of stone formation. Latexin (LXN) is reported to be downregulated in several types of tumors (67), and overexpression of LXN inhibits tumor cell growth (52, 67–69). LXN is also implicated in inflammation because it is highly enriched in mast cells (70) and can be upregulated by lipopolysaccharide (20, 71). The cellular senescence induced by overexpression of LXN was logically consistent with the suppressive effect of LXN on tumors (70) and its proinflammatory effect (21). The beneficial effect of LXN-mediated suppression was observed in this study, suggesting that the functional diversity of LXN might be involved in physiological or even pathogenic conditions and that different interventions should be considered based on the specific tissue or disease context (72). Thus further research is needed to harness its therapeutic potential.

In addition, alteration of the nuclear translocation of Rps3 and its downstream p53 expression after LXN suppression in our study were consistent with reports that ribosomal protein subunit 3 (Rps3) is a novel LXN-binding protein (60, 72, 73) and that p53 might be a downstream target of Rps3 (29, 74). Results of our study also aligned with previous report that the LXN–Rps3–p53 axis represented a critical regulatory network linking ribosomal stress via the RP–MDM2 pathway (29) and tumor suppression (75). Involvement of LXN–Rps3–p53 axis in oxidative stress adaptation (29) was further confirmed in our detection of oxidative stress indicators in cellular assays, and secondary cellular senescence was verified and comprehensively evaluated, suggesting the reliable effect of LXN on cellular senescence (76). Our research elucidates the impact of the LXN–Rps3–p53 axis on cellular senescence, providing the first evidence of its involvement in this biological process. More interestingly, p53 might also upregulate LXN expression (77), creating a negative feedback loop to restore ribosomal homeostasis (78). Therefore, targeting LXN, such as LXN inhibitors via high-throughput screening (79), nanoparticle delivery of LXN inhibitors, co-administration of LXN inhibitors and senolytics (e.g. Fisetin) could provide an effective treatment approach for cellular senescence-associated disorders. Furthermore, LXN may serve as a critical biomarker to determine whether to initiate anti-cellular senescence therapy (80).

The involvement of renal and peripheral macrophages in inflammatory processes might lead to the development of therapeutic targets (81, 82). With respect to the current consensus of macrophage involvement in CaOx nephrolithiasis (83), targeting M2-like macrophage function might offer a therapeutic strategy with which to prevent stones via crystal phagocytosis (83). In current macrophage research, TNF-α (84) and iNOS (53) are widely utilized as biomarkers for M1 macrophages, while Arg-1 (54) and CD163 (55) are commonly employed as M2 macrophage markers (56). Accordingly, in line with conventional macrophage classification schemes (57, 58), we utilized TNF-α/iNOS for M1 macrophages identification and Arg-1/CD163 for M2 macrophages characterization. To our knowledge, most studies have focused on intervening in effector macrophage polarization to suppress renal crystal deposition (11, 85); for example, Sirtuin 3 was found to suppress the formation of renal calcium oxalate crystals by promoting M2 polarization of macrophages (86); the critical role of the microenvironment in regulating macrophage function warrants further in-depth investigation (87). Our study focused on the origin of the shift in macrophage polarization and on the main cells of Randall’s plaques, RTECs (88), revealing that the cellular senescence of RTECs induced by calcium oxalate crystals was at least one of the factors that led to the suppression of M2 macrophage polarization, as opposed to the promotion of M1 macrophage polarization. More importantly, a context-dependent macrophage phenotype can also exacerbate the senescence of surrounding RTECs (89), resulting in a vicious cycle of kidney stone formation (66). Therefore, our findings reveal one possible mechanism by which reducing M2 macrophage polarization promotes the formation of kidney stones (83), undoubtedly providing a potential therapeutic and preventive approach to CaOx nephrolithiasis.

Senescence is a cellular program that involves changes in metabolism and the production of a bioactive secretome (90, 91). Our results suggest that this secretome, the senescence-associated secretory phenotype (SASP), mediates the recruitment and promotion of M1 macrophage polarization. The exact mechanism of macrophage–senescent cell interplay and its impact on macrophage effector functions during pathological conditions are extremely complex (92). The exact nature of macrophage polarization following recruitment to senescent cells *in vivo* is likely context dependent (92, 93). p53-expressing senescent stellate cells release factors that skew macrophage polarization toward a cytotoxic M1 state (94), whereas senescent thyrocytes induce M2-like polarization in human monocytes (95). Our research provides a more comprehensive understanding of macrophage–senescent cell interactions, and further research should be conducted to reveal more comprehensive and in-depth mechanisms, such as extracellular vesicle (EV) release (96), in addition to the SASP.

**Figure 7:**
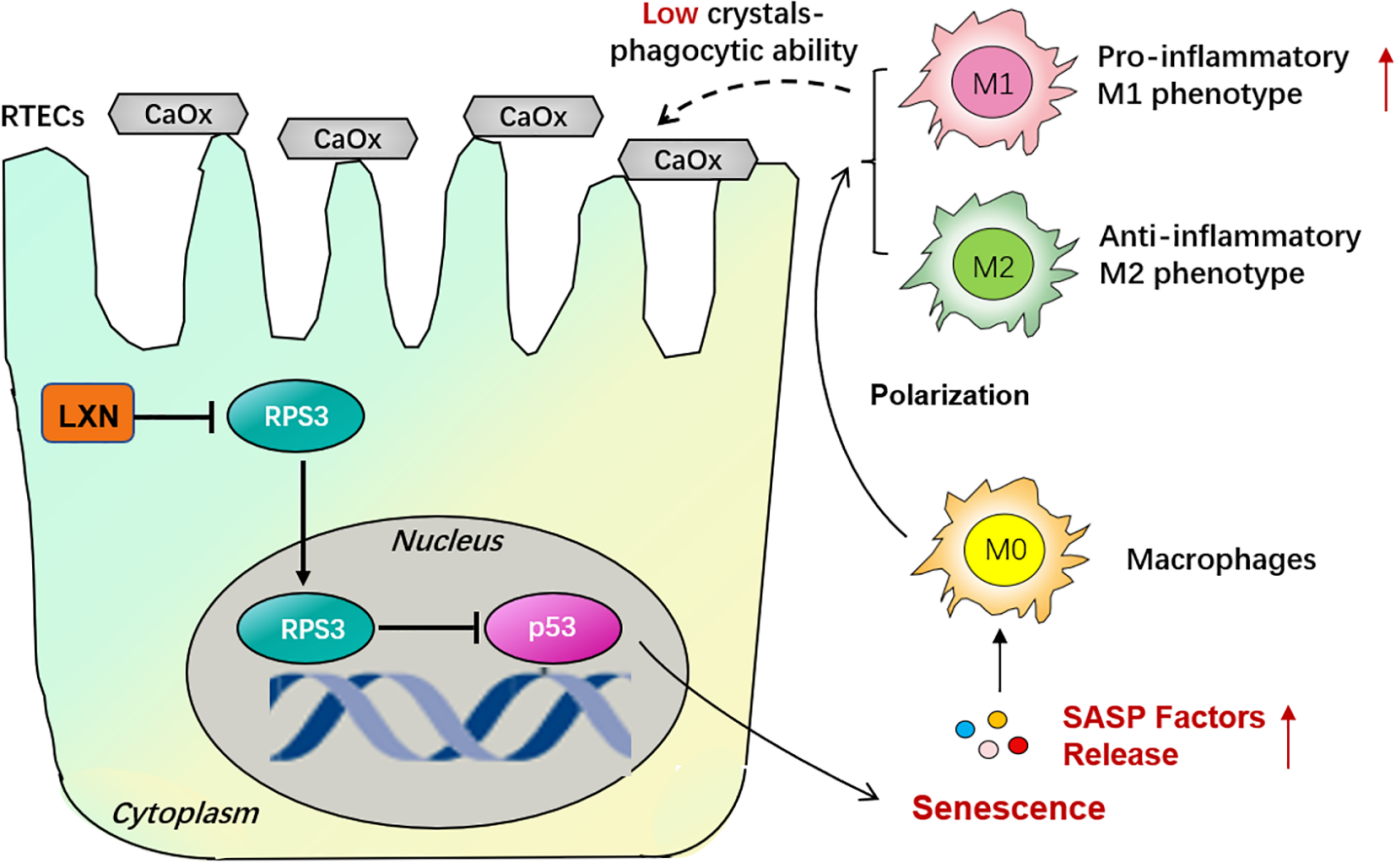
Schematic illustration of the mechanism of LXN/Rps3/p53 pathway on cellular senescence in CaOx stone formation. Activation of LXN gene in renal tubular epithelial cells (RTECs) is evoked by hyperoxaluria and oxalate. Since ribosomal protein subunit 3 (Rps3) is a LXN binding protein, the nuclear translocation of Rps3 were significantly down-regulated due to the more combination conducted by LXN protein. And then p53 which might be a downstream target of Rps3 is up-regulated, with a consequence of triggering RTECs’ senescence. Senescence-Associated Secretory Phenotype (SASP) factors are then released, skewing macrophage polarization towards pro-inflammatory M1 phenotype, not anti-inflammatory M2 phenotype, demonstrating a low crystals phagocytic ability. Therefore LXN gene targeting silencing could effectively inhibit cellular senescence, thus reduce macrophage polarization towards M1 phenotype and ultimately behave a protective effect on kidney stone formation.

Taken together, our results demonstrate that kidney M1-like phenotype macrophage polarization is associated with renal tubular epithelial cell senescence in oxalate-induced kidney stones and that the LXN/Rps3/p53 signaling pathway significantly decreases RTEC senescence-related M1-like phenotype macrophage polarization and ultimately alleviates intrarenal CaOx crystal deposition (**Figure 7**). Thus, our study provides a promising therapeutic target for the development of effective treatments for nephrolithiasis and other age-related diseases.

## Data Availability

The original contributions presented in the study are included in the article/**Supplementary Material**. Further inquiries can be directed to the corresponding authors.

## References

[1] Romero V Akpinar H Assimos DG . Kidney stones: a global picture of prevalence, incidence, and associated risk factors. Rev Urol. (2010) 12:e86–96., PMID: 20811557 PMC2931286

[2] Gault MH Chafe L . Relationship of frequency, age, sex, stone weight and composition in 15,624 stones: comparison of resutls for 1980 to 1983 and 1995 to 1998. J urology. (2000) 164:302–7. doi: 10.1016/S0022-5347(05)67345-4, PMID: 10893570

[3] Lieske JC Rule AD Krambeck AE Williams JC Bergstralh EJ Mehta RA . Stone composition as a function of age and sex. Clin J Am Soc Nephrology: CJASN. (2014) 9:2141–6. doi: 10.2215/cjn.05660614, PMID: 25278549 PMC4255407

[4] Khan SR . Reactive oxygen species as the molecular modulators of calcium oxalate kidney stone formation: evidence from clinical and experimental investigations. J urology. (2013) 189:803–11. doi: 10.1016/j.juro.2012.05.078, PMID: 23022011 PMC5683176

[5] Khan SR Canales BK Dominguez-Gutierrez PR . Randall's plaque and calcium oxalate stone formation: role for immunity and inflammation. Nat Rev Nephrology. (2021) 17:417–33. doi: 10.1038/s41581-020-00392-1, PMID: 33514941

[6] Grover PK Thurgood LA Fleming DE van Bronswijk W Wang T Ryall RL . Intracrystalline urinary proteins facilitate degradation and dissolution of calcium oxalate crystals in cultured renal cells. Am J Physiol Renal Physiol. (2008) 294:F355–61. doi: 10.1152/ajprenal.00529.2007, PMID: 18077596

[7] Taguchi K Okada A Kitamura H Yasui T Naiki T Hamamoto S . Colony-stimulating factor-1 signaling suppresses renal crystal formation. J Am Soc Nephrology: JASN. (2014) 25:1680–97. doi: 10.1681/asn.2013060675, PMID: 24578130 PMC4116057

[8] Kusmartsev S Dominguez-Gutierrez PR Canales BK Bird VG Vieweg J Khan SR . Calcium oxalate stone fragment and crystal phagocytosis by human macrophages. J urology. (2016) 195:1143–51. doi: 10.1016/j.juro.2015.11.048, PMID: 26626217 PMC4882284

[9] Vervaet BA Verhulst A Dauwe SE De Broe ME D'Haese PC . An active renal crystal clearance mechanism in rat and man. Kidney Int. (2009) 75:41–51. doi: 10.1038/ki.2008.450, PMID: 18784645

[10] Zhu W Zhao Z Chou F Zuo L Liu T Yeh S . Loss of the androgen receptor suppresses intrarenal calcium oxalate crystals deposition via altering macrophage recruitment/M2 polarization with change of the miR-185-5p/CSF-1 signals. Cell Death disease. (2019) 10:275. doi: 10.1038/s41419-019-1358-y, PMID: 30894518 PMC6427030

[11] Taguchi K Okada A Hamamoto S Unno R Moritoki Y Ando R . M1/M2-macrophage phenotypes regulate renal calcium oxalate crystal development. Sci Rep. (2016) 6:35167. doi: 10.1038/srep35167, PMID: 27731368 PMC5059697

[12] Taguchi K Okada A Hamamoto S Iwatsuki S Naiki T Ando R . Proinflammatory and metabolic changes facilitate renal crystal deposition in an obese mouse model of metabolic syndrome. J urology. (2015) 194:1787–96. doi: 10.1016/j.juro.2015.07.083, PMID: 26192255

[13] Kumari R Jat P . Mechanisms of cellular senescence: cell cycle arrest and senescence associated secretory phenotype. Front Cell Dev Biol. (2021) 9:645593. doi: 10.3389/fcell.2021.645593, PMID: 33855023 PMC8039141

[14] Mou Z Feng Z Xu Z Zhuang F Zheng X Li X . Schisandrin B alleviates diabetic nephropathy through suppressing excessive inflammation and oxidative stress. Biochem Biophys Res Commun. (2019) 508:243–9. doi: 10.1016/j.bbrc.2018.11.128, PMID: 30477745

[15] Coppé JP Desprez PY Krtolica A Campisi J . The senescence-associated secretory phenotype: the dark side of tumor suppression. Annu Rev Pathol. (2010) 5:99–118. doi: 10.1146/annurev-pathol-121808-102144, PMID: 20078217 PMC4166495

[16] Stanbury DM . The principle of detailed balancing, the iron-catalyzed disproportionation of hydrogen peroxide, and the Fenton reaction. Dalton Trans (Cambridge England: 2003). (2022) 51:2135–57. doi: 10.1039/d1dt03645a, PMID: 35029613

[17] Toussaint O Medrano EE von Zglinicki T . Cellular and molecular mechanisms of stress-induced premature senescence (SIPS) of human diploid fibroblasts and melanocytes. Exp gerontology. (2000) 35:927–45. doi: 10.1016/s0531-5565(00)00180-7, PMID: 11121681

[18] Sabin RJ Anderson RM . Cellular Senescence - its role in cancer and the response to ionizing radiation. Genome integrity. (2011) 2:7. doi: 10.1186/2041-9414-2-7, PMID: 21834983 PMC3169443

[19] Sun Y Kang J Guan X Xu H Wang X Deng Y . Regulation of endoplasmic reticulum stress on the damage and apoptosis of renal tubular epithelial cells induced by calcium oxalate crystals. Urolithiasis. (2021) 49:291–9. doi: 10.1007/s00240-021-01261-7, PMID: 33786645

[20] Aagaard A Listwan P Cowieson N Huber T Ravasi T Wells CA . An inflammatory role for the mammalian carboxypeptidase inhibitor latexin: relationship to cystatins and the tumor suppressor TIG1. Structure (London England: 1993). (2005) 13:309–17. doi: 10.1016/j.str.2004.12.013, PMID: 15698574

[21] Li Y Huang B Yang H Kan S Yao Y Liu X . Latexin deficiency in mice up-regulates inflammation and aggravates colitis through HECTD1/Rps3/NF-κB pathway. Sci Rep. (2020) 10:9868. doi: 10.1038/s41598-020-66789-x, PMID: 32555320 PMC7299958

[22] Kan S Li R Tan Y Yang F Xu S Wang L . Latexin deficiency attenuates adipocyte differentiation and protects mice against obesity and metabolic disorders induced by high-fat diet. Cell Death disease. (2022) 13:175. doi: 10.1038/s41419-022-04636-9, PMID: 35210404 PMC8873487

[23] Seed RI Taurozzi AJ Wilcock DJ Nappo G Erb HHH Read ML . The putative tumour suppressor protein Latexin is secreted by prostate luminal cells and is downregulated in Malignancy. Sci Rep. (2019) 9:5120. doi: 10.1038/s41598-019-41379-8, PMID: 30914656 PMC6435711

[24] Wei Z Chen XC Song Y Pan XD Dai XM Zhang J . Amyloid β Protein aggravates neuronal senescence and cognitive deficits in 5XFAD mouse model of alzheimer's disease. Chin Med J. (2016) 129:1835–44. doi: 10.4103/0366-6999.186646, PMID: 27453234 PMC4976573

[25] Zha Y Zhuang W Yang Y Zhou Y Li H Liang J . Senescence in vascular smooth muscle cells and atherosclerosis. Front Cardiovasc Med. (2022) 9:910580. doi: 10.3389/fcvm.2022.910580, PMID: 35722104 PMC9198250

[26] Wang J Wang Z Wang H Wanyan Z Pan Y Zhu F . Stress-induced premature senescence promotes proliferation by activating the SENEX and p16(INK4a)/retinoblastoma (Rb) pathway in diffuse large B-cell lymphoma. Turkish J haematology: Off J Turkish Soc Haematology. (2019) 36:247–54. doi: 10.4274/tjh.galenos.2019.2019.0117, PMID: 31327185 PMC6863019

[27] Zhou Y Höti N Ao M Zhang Z Zhu H Li L . Expression of p16 and p53 in non-small-cell lung cancer: clinicopathological correlation and potential prognostic impact. Biomarkers Med. (2019) 13:761–71. doi: 10.2217/bmm-2018-0441, PMID: 31157548 PMC8173521

[28] He G Ni Y Hua R Wan H Tan Y Chen Q . Latexin deficiency limits foam cell formation and ameliorates atherosclerosis by promoting macrophage phenotype differentiation. Cell Death disease. (2024) 15:754. doi: 10.1038/s41419-024-07141-3, PMID: 39424784 PMC11492231

[29] Yadavilli S Mayo LD Higgins M Lain S Hegde V Deutsch WA . Ribosomal protein S3: A multi-functional protein that interacts with both p53 and MDM2 through its KH domain. DNA repair. (2009) 8:1215–24. doi: 10.1016/j.dnarep.2009.07.003, PMID: 19656744 PMC2748156

[30] Saleh T Carpenter VJ Tyutyunyk-Massey L Murray G Leverson JD Souers AJ . Clearance of therapy-induced senescent tumor cells by the senolytic ABT-263 via interference with BCL-X(L) -BAX interaction. Mol Oncol. (2020) 14:2504–19. doi: 10.1002/1878-0261.12761, PMID: 32652830 PMC7530780

[31] Du Y Huang F Guan L Zeng M . Role of PI3K/Akt/mTOR pathway-mediated macrophage autophagy in affecting the phenotype transformation of lung fibroblasts induced by silica dust exposure. Zhong nan da xue xue bao Yi xue ban = J Cent South Univ Med Sci. (2023) 48:1152–62. doi: 10.11817/j.issn.1672-7347.2023.220581, PMID: 37875355 PMC10930851

[32] Kim YK Hwang JH Lee HT . Differential susceptibility to lipopolysaccharide affects the activation of toll-like-receptor 4 signaling in THP-1 cells and PMA-differentiated THP-1 cells. Innate Immun. (2022) 28:122–9. doi: 10.1177/17534259221100170, PMID: 35612375 PMC9136465

[33] Smith MP Young H Hurlstone A Wellbrock C . Differentiation of THP1 cells into macrophages for transwell co-culture assay with melanoma cells. Bio-protocol. (2015) 5(21):e1638. doi: 10.21769/bioprotoc.1638, PMID: 27034969 PMC4811304

[34] Scott TE Lewis CV Zhu M Wang C Samuel CS Drummond GR . IL-4 and IL-13 induce equivalent expression of traditional M2 markers and modulation of reactive oxygen species in human macrophages. Sci Rep. (2023) 13:19589. doi: 10.1038/s41598-023-46237-2, PMID: 37949903 PMC10638413

[35] Joshi H Anaya E Addanki A Almgren-Bell A Todd EM Morley SC . Mechanosensitivity of macrophage polarization: comparing small molecule leukadherin-1 to substrate stiffness. Front Immunol. (2025) 16:1420325. doi: 10.3389/fimmu.2025.1420325, PMID: 40114914 PMC11922956

[36] Rayess H Wang MB Srivatsan ES . Cellular senescence and tumor suppressor gene p16. Int J cancer. (2012) 130:1715–25. doi: 10.1002/ijc.27316, PMID: 22025288 PMC3288293

[37] Konecny GE Winterhoff B Kolarova T Qi J Manivong K Dering J . Expression of p16 and retinoblastoma determines response to CDK4/6 inhibition in ovarian cancer. Clin Cancer research: an Off J Am Assoc Cancer Res. (2011) 17:1591–602. doi: 10.1158/1078-0432.Ccr-10-2307, PMID: 21278246 PMC4598646

[38] Goel S DeCristo MJ McAllister SS Zhao JJ . CDK4/6 inhibition in cancer: beyond cell cycle arrest. Trends Cell Biol. (2018) 28:911–25. doi: 10.1016/j.tcb.2018.07.002, PMID: 30061045 PMC6689321

[39] Hinkal GW Gatza CE Parikh N Donehower LA . Altered senescence, apoptosis, and DNA damage response in a mutant p53 model of accelerated aging. Mech Ageing Dev. (2009) 130:262–71. doi: 10.1016/j.mad.2009.01.001, PMID: 19396980 PMC2722837

[40] Pitolli C Wang Y Candi E Shi Y Melino G Amelio I . p53-mediated tumor suppression: DNA-damage response and alternative mechanisms. Cancers. (2019) 11(12):1983. doi: 10.3390/cancers11121983, PMID: 31835405 PMC6966539

[41] Zhang J Zhang Y Ma Y Luo L Chu M Zhang Z . Therapeutic Potential of Exosomal circRNA Derived from Synovial Mesenchymal Cells via Targeting circEDIL3/miR-485-3p/PIAS3/STAT3/VEGF Functional Module in Rheumatoid Arthritis. Int J nanomedicine. (2021) 16:7977–94. doi: 10.2147/ijn.S333465, PMID: 34887661 PMC8651050

[42] Zhang J Ma Y Zhang Y Niu S Chu M Zhang Z . Angiogenesis is inhibited by arsenic trioxide through downregulation of the circHIPK3/miR-149-5p/FOXO1/VEGF functional module in rheumatoid arthritis. Front Pharmacol. (2021) 12:751667. doi: 10.3389/fphar.2021.751667, PMID: 34776969 PMC8579003

[43] Lin Z Ma Y Zhu X Dai S Sun W Li W . Potential predictive and therapeutic applications of small extracellular vesicles-derived circPARD3B in osteoarthritis. Front Pharmacol. (2022) 13:968776. doi: 10.3389/fphar.2022.968776, PMID: 36339585 PMC9627215

[44] Yang B Wang G Li Y Yang T Guo H Li P . Hydroxycitric acid prevents hyperoxaluric-induced nephrolithiasis and oxidative stress via activation of the Nrf2/Keap1 signaling pathway. Cell Cycle (Georgetown Tex). (2023) 22:1884–99. doi: 10.1080/15384101.2023.2247251, PMID: 37592762 PMC10599177

[45] Ijima S Saito Y Nagaoka K Yamamoto S Sato T Miura N . Fisetin reduces the senescent tubular epithelial cell burden and also inhibits proliferative fibroblasts in murine lupus nephritis. Front Immunol. (2022) 13:960601. doi: 10.3389/fimmu.2022.960601, PMID: 36466895 PMC9714549

[46] Jing G-H Liu Y-D Liu J-N Jin Y-S Yu S-L An R-H . Puerarin prevents calcium oxalate crystal-induced renal epithelial cell autophagy by activating the SIRT1-mediated signaling pathway. Urolithiasis. (2022) 50:545–56. doi: 10.1007/s00240-022-01347-w, PMID: 35913552

[47] Lin Z Li W Wang Y Lang X Sun W Zhu X . SMSCs-derived sEV overexpressing miR-433-3p inhibits angiogenesis induced by sEV released from synoviocytes under triggering of ferroptosis. Int Immunopharmacol. (2023) 116:109875. doi: 10.1016/j.intimp.2023.109875, PMID: 37501360

[48] Ma Y Li W Niu S Zhu X Chu M Wang W . BzATP reverses ferroptosis-induced gut microbiota disorders in collagen-induced arthritis mice. Int Immunopharmacol. (2023) 124:110885. doi: 10.1016/j.intimp.2023.110885, PMID: 37713784

[49] Chu M Zhang C . Inhibition of angiogenesis by leflunomide via targeting the soluble ephrin-A1/EphA2 system in bladder cancer. Sci Rep. (2018) 8:1539. doi: 10.1038/s41598-018-19788-y, PMID: 29367676 PMC5784165

[50] Dong Z Chen F Peng S Liu X Liu X Guo L . Identification of the key immune-related genes and immune cell infiltration changes in renal interstitial fibrosis. Front endocrinology. (2023) 14:1207444. doi: 10.3389/fendo.2023.1207444, PMID: 38027143 PMC10663291

[51] Zhang X Li Y Ma Z He D Li H . Modulating degradation of sodium alginate/bioglass hydrogel for improving tissue infiltration and promoting wound healing. Bioactive materials. (2021) 6:3692–704. doi: 10.1016/j.bioactmat.2021.03.038, PMID: 33898873 PMC8056275

[52] Muthusamy V Premi S Soper C Platt J Bosenberg M . The hematopoietic stem cell regulatory gene latexin has tumor-suppressive properties in Malignant melanoma. J Invest Dermatol. (2013) 133:1827–33. doi: 10.1038/jid.2013.48, PMID: 23364479 PMC3683103

[53] Rath M Müller I Kropf P Closs EI Munder M . Metabolism via arginase or nitric oxide synthase: two competing arginine pathways in macrophages. Front Immunol. (2014) 5:532. doi: 10.3389/fimmu.2014.00532, PMID: 25386178 PMC4209874

[54] Pesce JT Ramalingam TR Mentink-Kane MM Wilson MS El Kasmi KC Smith AM . Arginase-1-expressing macrophages suppress Th2 cytokine-driven inflammation and fibrosis. PloS pathogens. (2009) 5:e1000371. doi: 10.1371/journal.ppat.1000371, PMID: 19360123 PMC2660425

[55] Etzerodt A Moestrup SK . CD163 and inflammation: biological, diagnostic, and therapeutic aspects. Antioxidants Redox Signaling. (2013) 18:2352–63. doi: 10.1089/ars.2012.4834, PMID: 22900885 PMC3638564

[56] Murray PJ Allen JE Biswas SK Fisher EA Gilroy DW Goerdt S . Macrophage activation and polarization: nomenclature and experimental guidelines. Immunity. (2014) 41:14–20. doi: 10.1016/j.immuni.2014.06.008, PMID: 25035950 PMC4123412

[57] Williams JW Giannarelli C Rahman A Randolph GJ Kovacic JC . Macrophage biology, classification, and phenotype in cardiovascular disease: JACC macrophage in CVD series (Part 1). J Am Coll Cardiol. (2018) 72:2166–80. doi: 10.1016/j.jacc.2018.08.2148, PMID: 30360826 PMC6209330

[58] Wei Y Wang M Ma Y Que Z Yao D . Classical dichotomy of macrophages and alternative activation models proposed with technological progress. BioMed Res Int. (2021) 2021:9910596. doi: 10.1155/2021/9910596, PMID: 34722776 PMC8553456

[59] Dong C Song C He Z Song Q Song T Liu J . Protective efficacy of Schizandrin B on ameliorating nephrolithiasis via regulating GSK3β/Nrf2 signaling-mediated ferroptosis *in vivo* and *in vitro*. Int Immunopharmacol. (2023) 117:110042. doi: 10.1016/j.intimp.2023.110042, PMID: 36940552

[60] You Y Wen R Pathak R Li A Li W St Clair D . Latexin sensitizes leukemogenic cells to gamma-irradiation-induced cell-cycle arrest and cell death through Rps3 pathway. Cell Death disease. (2014) 5:e1493. doi: 10.1038/cddis.2014.443, PMID: 25341047 PMC4237263

[61] Edelmann MJ Shack LA Naske CD Walters KB Nanduri B . SILAC-based quantitative proteomic analysis of human lung cell response to copper oxide nanoparticles. PloS One. (2014) 9:e114390. doi: 10.1371/journal.pone.0114390, PMID: 25470785 PMC4255034

[62] Frassetto L Kohlstadt I . Treatment and prevention of kidney stones: an update. Am Family physician. (2011) 84:1234–42., PMID: 22150656

[63] Moudi E Hosseini SR Bijani A . Nephrolithiasis in elderly population; effect of demographic characteristics. J nephropathology. (2017) 6:63–8. doi: 10.15171/jnp.2017.11, PMID: 28491855 PMC5418072

[64] Palmer AK Gustafson B Kirkland JL Smith U . Cellular senescence: at the nexus between ageing and diabetes. Diabetologia. (2019) 62:1835–41. doi: 10.1007/s00125-019-4934-x, PMID: 31451866 PMC6731336

[65] Tuttle CSL Waaijer MEC Slee-Valentijn MS Stijnen T Westendorp R Maier AB . Cellular senescence and chronological age in various human tissues: A systematic review and meta-analysis. Aging Cell. (2020) 19:e13083. doi: 10.1111/acel.13083, PMID: 31808308 PMC6996941

[66] Chuenwisad K More-Krong P Tubsaeng P Chotechuang N Srisa-Art M Storer RJ . Premature senescence and telomere shortening induced by oxidative stress from oxalate, calcium oxalate monohydrate, and urine from patients with calcium oxalate nephrolithiasis. Front Immunol. (2021) 12:696486. doi: 10.3389/fimmu.2021.696486, PMID: 34745087 PMC8566732

[67] Li Y Basang Z Ding H Lu Z Ning T Wei H . Latexin expression is downregulated in human gastric carcinomas and exhibits tumor suppressor potential. BMC cancer. (2011) 11:121. doi: 10.1186/1471-2407-11-121, PMID: 21466706 PMC3080345

[68] Abd Elmageed ZY Moroz K Kandil E . Clinical significance of CD146 and latexin during different stages of thyroid cancer. Mol Cell Biochem. (2013) 381:95–103. doi: 10.1007/s11010-013-1691-x, PMID: 23712706

[69] Ni QF Tian Y Kong LL Lu YT Ding WZ Kong LB . Latexin exhibits tumor suppressor potential in hepatocellular carcinoma. Oncol Rep. (2014) 31:1364–72. doi: 10.3892/or.2014.2966, PMID: 24399246

[70] Xue Z Zhou Y Wang C Zheng J Zhang P Zhou L . Latexin exhibits tumor-suppressor potential in pancreatic ductal adenocarcinoma. Oncol Rep. (2016) 35:50–8. doi: 10.3892/or.2015.4353, PMID: 26530530 PMC4699618

[71] Uratani Y Takiguchi-Hayashi K Miyasaka N Sato M Jin M Arimatsu Y . Latexin, a carboxypeptidase A inhibitor, is expressed in rat peritoneal mast cells and is associated with granular structures distinct from secretory granules and lysosomes. Biochem J. (2000) 346 Pt 3:817–26. doi: 10.1042/bj3460817, PMID: 10698712 PMC1220918

[72] Sarsenova M Stepanjuk A Saare M Kasvandik S Soplepmann P Mikeltadze I . Carboxypeptidase inhibitor LXN expression in endometrial tissue is menstrual cycle phase-dependent and is upregulated in endometriotic lesions. Genes. (2024) 15(8):1086. doi: 10.3390/genes15081086, PMID: 39202445 PMC11353285

[73] Zhang C Cui X Liu Y Wang F Signer R Nattamai K . Latexin deletion protects against radiation-induced hematopoietic damages via selective activation of Bcl-2 prosurvival pathway. Haematologica. (2023) 108:3464–70. doi: 10.3324/haematol.2022.282028, PMID: 37345464 PMC10690908

[74] Alam E Maaliki L Nasr Z . Ribosomal protein S3 selectively affects colon cancer growth by modulating the levels of p53 and lactate dehydrogenase. Mol Biol Rep. (2020) 47:6083–90. doi: 10.1007/s11033-020-05683-1, PMID: 32748020

[75] Cui G . Immune battle at the premalignant stage of colorectal cancer: focus on immune cell compositions, functions and cytokine products. Am J Cancer Res. (2020) 10:1308–20., PMID: 32509381 PMC7269793

[76] Liu B Peng Z Zhang H Zhang N Liu Z Xia Z . Regulation of cellular senescence in tumor progression and therapeutic targeting: mechanisms and pathways. Mol cancer. (2025) 24:106. doi: 10.1186/s12943-025-02284-z, PMID: 40170077 PMC11963325

[77] Lee SY Jang Y Seok HY Moon YH . A novel mechanism of the p53 isoform Δ40p53α in regulating collagen III expression in TGFβ1-induced LX-2 human hepatic stellate cells. FASEB journal: Off Publ Fed Am Societies Exp Biol. (2025) 39:e70541. doi: 10.1096/fj.202403146RR, PMID: 40232888 PMC11999059

[78] Zisi A Bartek J Lindström MS . Targeting ribosome biogenesis in cancer: lessons learned and way forward. Cancers. (2022) 14(9):2126. doi: 10.3390/cancers14092126, PMID: 35565259 PMC9100539

[79] Zielonka J Zielonka M Cheng G Hardy M Kalyanaraman B . High-throughput screening of NOX inhibitors. Methods Mol Biol (Clifton NJ). (2019) 1982:429–46. doi: 10.1007/978-1-4939-9424-3_25, PMID: 31172487 PMC6953630

[80] Czajkowski K Herbet M Murias M Piątkowska-Chmiel I . Senolytics: charting a new course or enhancing existing anti-tumor therapies? Cell Oncol (Dordrecht Netherlands). (2025) 48:351–71. doi: 10.1007/s13402-024-01018-5, PMID: 39633108 PMC11996976

[81] Islamuddin M Qin X . Renal macrophages and NLRP3 inflammasomes in kidney diseases and therapeutics. Cell Death Discov. (2024) 10:229. doi: 10.1038/s41420-024-01996-3, PMID: 38740765 PMC11091222

[82] Duffield JS . Macrophages and immunologic inflammation of the kidney. Semin nephrology. (2010) 30:234–54. doi: 10.1016/j.semnephrol.2010.03.003, PMID: 20620669 PMC2922007

[83] Taguchi K Okada A Unno R Hamamoto S Yasui T . Macrophage function in calcium oxalate kidney stone formation: A systematic review of literature. Front Immunol. (2021) 12:673690. doi: 10.3389/fimmu.2021.673690, PMID: 34108970 PMC8182056

[84] Pérez S Rius-Pérez S . Macrophage polarization and reprogramming in acute inflammation: A redox perspective. Antioxidants (Basel Switzerland). (2022) 11(7):1394. doi: 10.3390/antiox11071394, PMID: 35883885 PMC9311967

[85] Lu H Sun X Jia M Sun F Zhu J Chen X . Rosiglitazone suppresses renal crystal deposition by ameliorating tubular injury resulted from oxidative stress and inflammatory response via promoting the nrf2/HO-1 pathway and shifting macrophage polarization. Oxid Med Cell longevity. (2021) 2021:5527137. doi: 10.1155/2021/5527137, PMID: 34691355 PMC8531781

[86] Xi J Chen Y Jing J Zhang Y Liang C Hao Z . Sirtuin 3 suppresses the formation of renal calcium oxalate crystals through promoting M2 polarization of macrophages. J Cell Physiol. (2019) 234:11463–73. doi: 10.1002/jcp.27803, PMID: 30588609

[87] Ham S Lima LG Lek E Möller A . The impact of the cancer microenvironment on macrophage phenotypes. Front Immunol. (2020) 11:1308. doi: 10.3389/fimmu.2020.01308, PMID: 32655574 PMC7324670

[88] Saleh LS Amer LD Thompson BJ Danhorn T Knapp JR Gibbings SL . Mapping Macrophage Polarization and Origin during the Progression of the Foreign Body Response to a Poly(ethylene glycol) Hydrogel Implant. Advanced healthcare materials. (2022) 11:e2102209. doi: 10.1002/adhm.202102209, PMID: 34967497 PMC9081184

[89] Liu Q Liu Y Guan X Wu J He Z Kang J . Effect of M2 macrophages on injury and apoptosis of renal tubular epithelial cells induced by calcium oxalate crystals. Kidney Blood Pressure Res. (2019) 44:777–91. doi: 10.1159/000501558, PMID: 31408871

[90] D'Ambrosio M Gil J . Reshaping of the tumor microenvironment by cellular senescence: An opportunity for senotherapies. Dev Cell. (2023) 58:1007–21. doi: 10.1016/j.devcel.2023.05.010, PMID: 37339603

[91] Frasca D Saada YB Garcia D Friguet B . Effects of cellular senescence on metabolic pathways in non-immune and immune cells. Mech Ageing Dev. (2021) 194:111428. doi: 10.1016/j.mad.2020.111428, PMID: 33383073 PMC7882031

[92] Behmoaras J Gil J . Similarities and interplay between senescent cells and macrophages. J Cell Biol. (2021) 220(2):e202010162. doi: 10.1083/jcb.202010162, PMID: 33355620 PMC7769159

[93] Elder SS Emmerson E . Senescent cells and macrophages: key players for regeneration? Open Biol. (2020) 10:200309. doi: 10.1098/rsob.200309, PMID: 33352064 PMC7776574

[94] Lujambio A Akkari L Simon J Grace D Tschaharganeh DF Bolden JE . Non-cell-autonomous tumor suppression by p53. Cell. (2013) 153:449–60. doi: 10.1016/j.cell.2013.03.020, PMID: 23562644 PMC3702034

[95] Mazzoni M Mauro G Erreni M Romeo P Minna E Vizioli MG . Senescent thyrocytes and thyroid tumor cells induce M2-like macrophage polarization of human monocytes via a PGE2-dependent mechanism. J Exp Clin Cancer research: CR. (2019) 38:208. doi: 10.1186/s13046-019-1198-8, PMID: 31113465 PMC6528237

[96] Loarca L De Assuncao TM Jalan-Sakrikar N Bronk S Krishnan A Huang B . Development and characterization of cholangioids from normal and diseased human cholangiocytes as an *in vitro* model to study primary sclerosing cholangitis. Lab investigation; J Tech Methods Pathol. (2017) 97:1385–96. doi: 10.1038/labinvest.2017.63, PMID: 28892096 PMC5664217

