# Correction: Premature renal epithelial cell senescence promoted by LXN/Rps3/p53 signaling pathway activation increases calcium oxalate crystal deposition by altering macrophage polarization

**DOI:** 10.3389/fimmu.2026.1790014

**Published:** 2026-05-05

**Authors:** Maolin Chu, Suna Jiang, Jiawei Xue, Wenjing Li, Guanhua Jing, Hongying Li, Juan Zhang, Wanhai Xu

**Affiliations:** 1 Department of Urology, The Second Affiliated Hospital, Harbin Medical University, Harbin, China; 2 Department of Rheumatology, The First Affiliated Hospital, Harbin Medical University, Harbin, China; 3 Department of Urology, Henan Provincial People’s Hospital (People’s Hospital of Zhengzhou University), Zhengzhou, China

**Keywords:** nephrolithiasis, cellular senescence, calcium oxalate, LXN, Rps3

File **Supplemental_Figure_5.tif** was erroneously published with the original version of this paper. The file has now been corrected.

The original version of this article has been updated.

