# Supplementary material for: Premature renal epithelial cell senescence promoted by LXN/Rps3/p53 signaling pathway activation increases calcium oxalate crystal deposition by altering macrophage polarization
Source: Front Immunol. 2025 Oct 2;16:1658989. doi: 10.3389/fimmu.2025.1658989 (PMC12527873; doi:10.3389/fimmu.2025.1658989)
Supplement: Supplementary file 1 [file DataSheet1.docx]

**
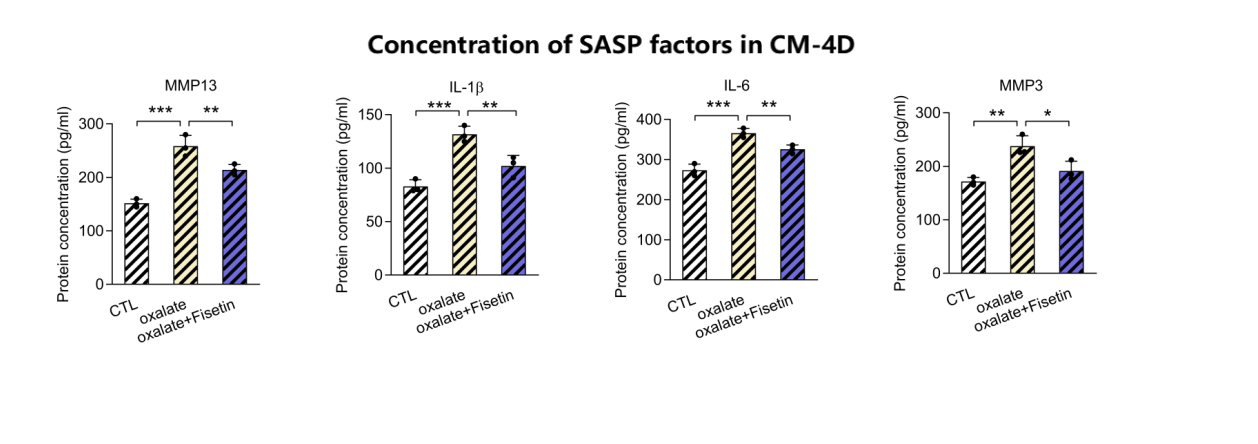
Supplementary Figure 1. Levels of SASP factors in CM-4D.**

HK-2 cells were incubated under 0.5 mM oxalate with or without 10 μM Fisetin for 2 days. On the third day, the supernatants were replaced by fresh culture medium, and 2 days later, the culture medium (CM-4D) were obtained. SASP factors, IL-1β, MMP13, IL-6 and MMP3 levels in CM-4D from HK-2 cells induced by oxalate were significantly up-regulated, and this up-regulation were significantly decreased in CM-4D from oxalate+Fisetin treated HK-2 cells culture. Based on three independent experiments. Data are presented as means ± SD. **P*< 0.05, ***P*< 0.01, ****P*< 0.001; CTL = control group.

**
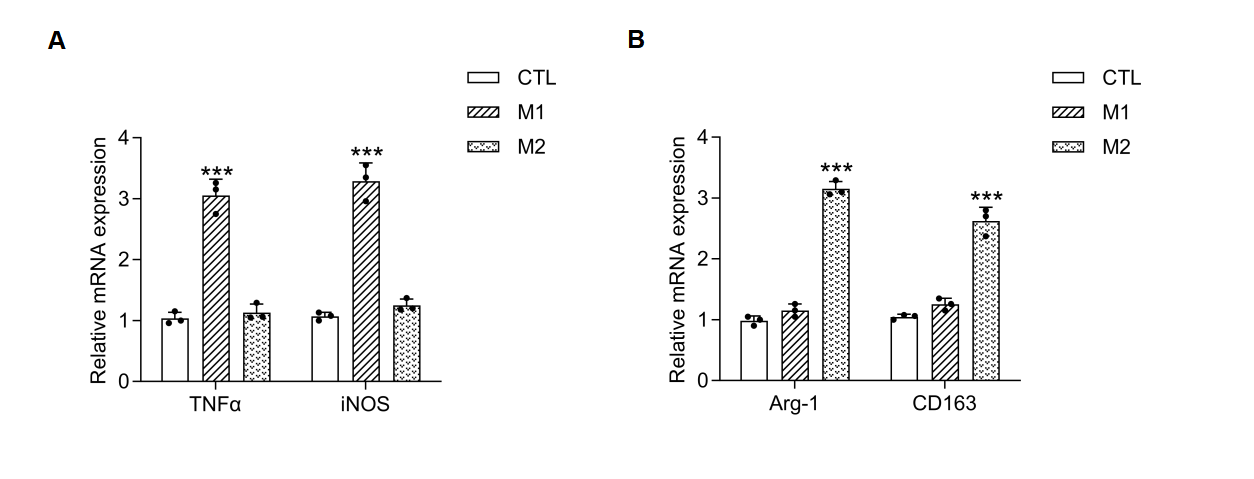
 Supplementary Figure 2. Relative mRNA expression of polarization markers in M1 and M2 macrophages.**

(A) Compared with native macrophages, the mRNA expression of TNF-α and iNOS were increased in M1 macrophages. (B) Expression of Arg-1 and CD63 mRNA were higher in M2 macrophages compared to native macrophages. Data are presented as means ± SD. ****P*< 0.001; CTL = control group.

**
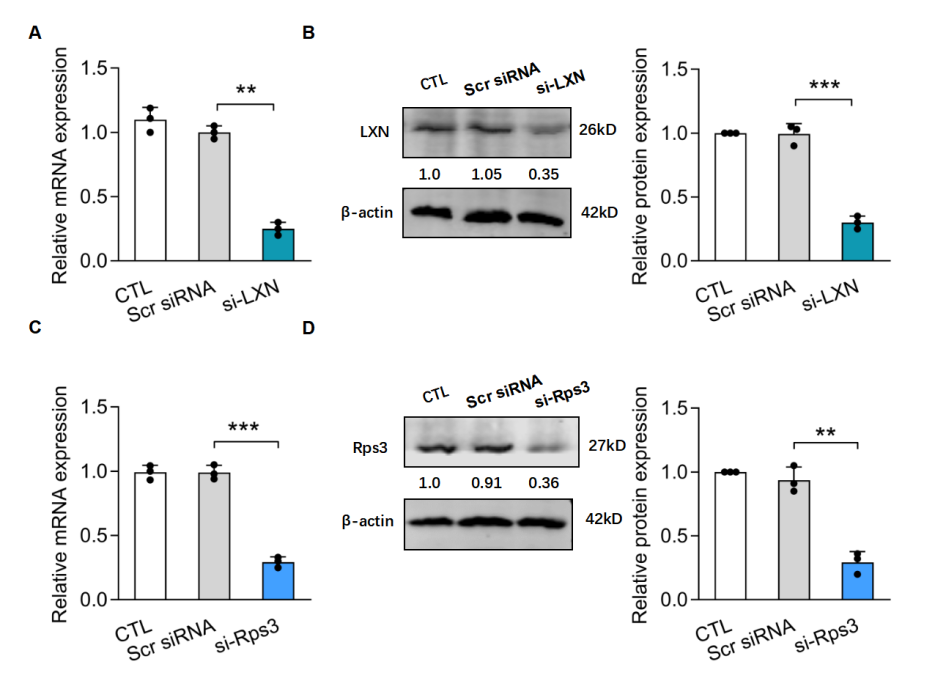
**

**Supplementary Figure 3. Knockdown efficiency of LXN or Rps3 gene.**

LXN/Rps3 knockdown in HK-2 cells were carefully performed, and the knockdown efficiency of LXN/Rps3 were respectively determined by RT-qPCR (A, C) and western blot analysis (B, D). At 48 h after transfection, 75% knockdown efficiency was achieved. Data are presented as means ± SD. ***P*< 0.01, ****P*< 0.001; CTL = control group.

**
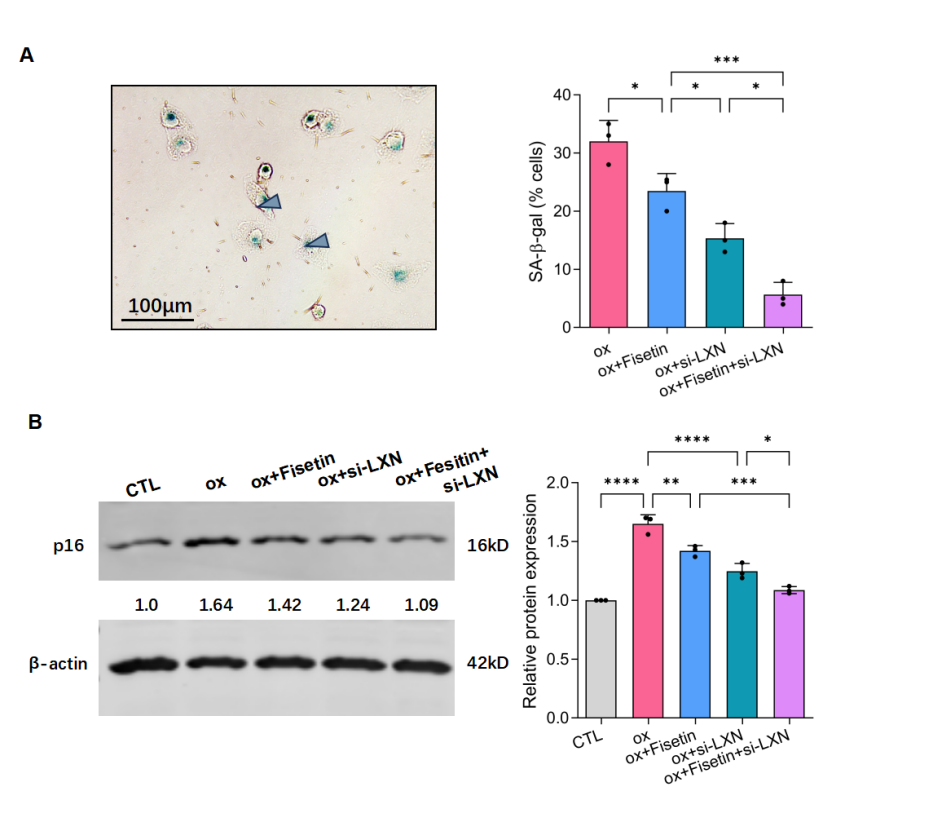
**

**Supplementary Figure 4.** **Cellular senescence levels in the combined treatment group of fisetin and LXN knockdown.** (A) Results of staining of HK-2 cells. (B) Detection results of p16 protein expression via western blot analysis. Data are presented as means ± SD. **P* < 0.05, ***P*< 0.01, ****P*< 0.001; CTL = control group.

**
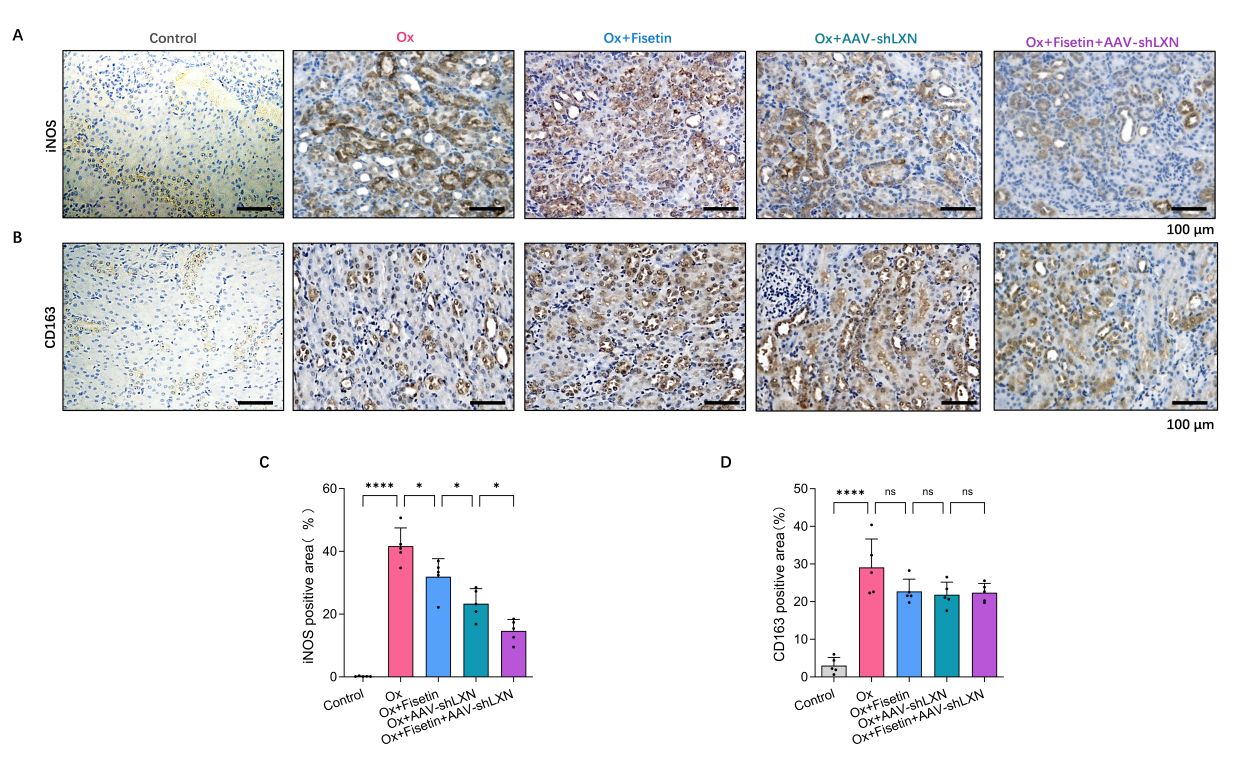
**

**Supplementary Figure 5. Targeting cellular senescence could ameliorate the renal abnormal macrophage polarization in the rat kidney stone model.**

Representative immunohistochemical staining images of iNOS (A) and CD163 (B) plus quantitative analysis of positive areas (C-D) in different treatment groups of rat renal calculus models. The scale bar represents 100 μm. Data are expressed as the mean ± SD. Significance levels: * *P* < 0.05, **** *P* < 0.0001. “ns” indicates no significant difference.

**
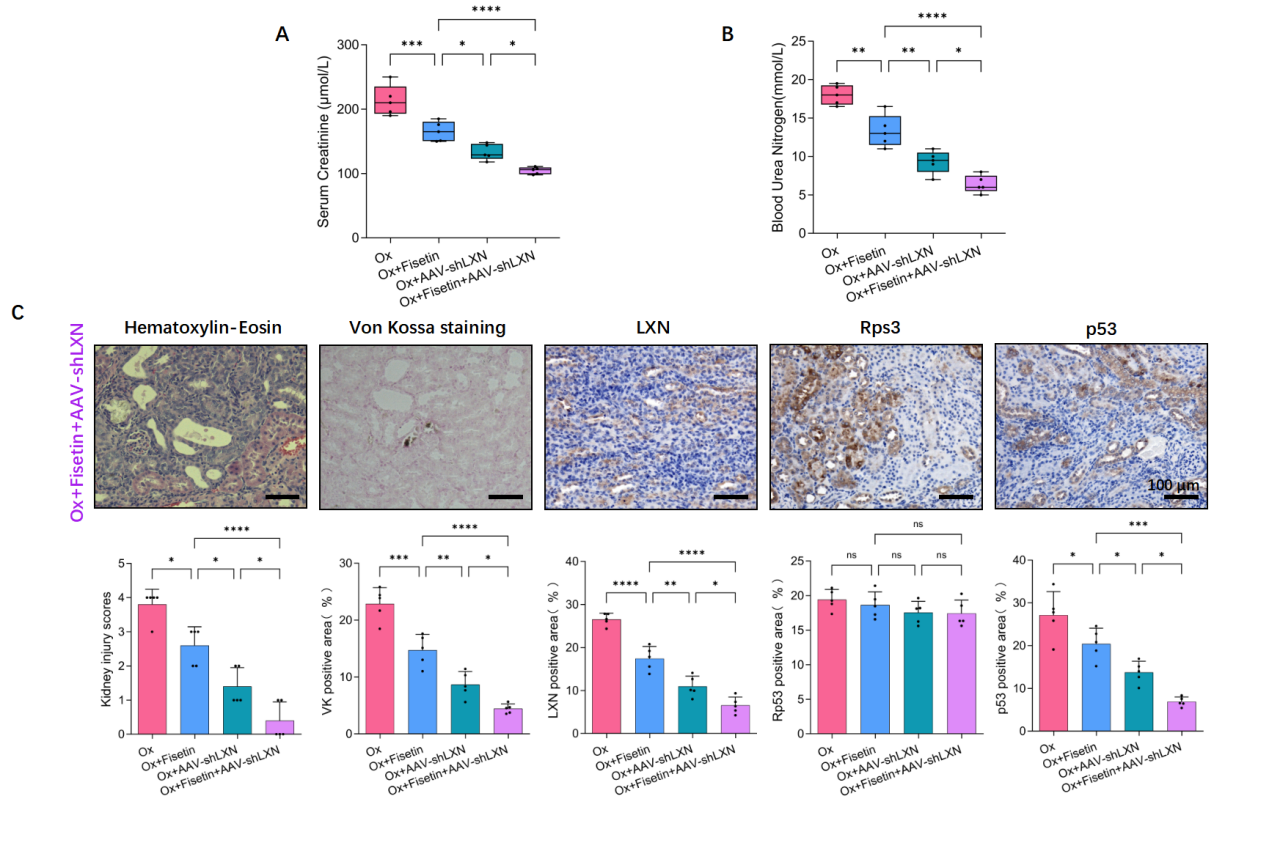
**

**Supplementary Figure 6. *In vivo* experimental results of combined treatment of fisetin and LXN knockdown.**

In renal calculi model rats, the levels of blood creatinine (A) and urea nitrogen (B) in the fisetin combined with AAV-sh-LXN treatment group were compared with those in other treatment groups. Representative images (C) of hematoxylin and eosin (H&E) staining, Von Kossa (VK) staining, and immunohistochemical staining for LXN, Rp53, and p53 and quantitative statistics (D) were shown. The scale bar represents 100 μm. Data are expressed as the mean ± SD. Significance levels: **P* < 0.05, ** *P* < 0.01, *** *P* < 0.001, *****P* < 0.0001. “ns” indicates no significant difference.

**Supplementary Table 1 (Table S 1). Primers and probes used in the study.**

| **Gene** | **Sequence** |
| --- | --- |
| VEGF | 5’–TTCTGGGCTGTTCTCGCTTC–3’  3’–CTCTCCTCTTCCTTCTCTTCTTCC–5’ |
| MMP13 | 5’–TGACTGGCAAACTTGAGACGATA–3’  3’–AGGGTGTAATCACCATCTGTAG–5’ |
| IL-1β | 5’–TGGCAGAAAGGGAACAGAA–3’  3’–ACAACAGGAAAGTCCAGGCTA–5’ |
| MCP-1 | 5’–AAGACCATTGTGGCCAAGGA–3’  3’–CGGAGTTTGGGTTTGCTTGT–5’ |
| iNOS | 5’-AGCCTGTGAGACGTTTGATGT-3’  5’-TGTAGATTCTGCCGAGATTTGA-3’ |
| Arg-1 | 5'-GGCTGGTCTGCTTGAGAAAC-3'  5'-ATTGCCAAACTGTGGTCTCC-3' |
| CD163 | 5’-CGGCTGCCTCCACCTCTAAGT-3′  5’-ATGAAGATGCTGGCGTGACA-3’ |
| LXN | 5′-CAGAGGAAACGCTGGACTCT-3′  3′-AGCCAGATTTGCTTGTTTGG-5′ |
| Rps3 | 5′-AGCGGAGACCCTGTTAACTACTAC-3′  3′-GTCTTTCTACAAAATTTTATTAAAGG-3′ |
| p53 | 5′-TCTGTCCCTTCCCAGAAAACC-3′  3′-CGTCATGTGCTGTGACTGCTT-5′ |
| GLS | 5′-GAGGCACCTTGGATAAGCTGGA-3′  3′-GCTGTCACATCTCTGGCTGCATA-5′ |
| PTGS1 | 5′-TGCGCTCCAACCTTATCCC-3′  3′-AGAGGGCAGAATACGAGTGTAA-5′ |
| CFB | 5′-CTGTGGCATGGTTTGG-3′  3′-GGGCGAATGACTGAGAT-5′ |
| GAPDH | 5′-GCACCGTCAAGGCTGAGAAC-3′  3′-TGGTGAAGACGCCAGTGGA-5′ |
| **siRNAs** |  |
| si-LXN | SASI_Hs01_00073945（Sigma-Aldrich） |
| si-Rps3 | SASI_Hs01_00243869（Sigma-Aldrich） |
| Scrambled control siRNA | MISSION siRNA Universal Negative Control（Sigma-Aldrich） |
